# Caspases maintain tissue integrity by an apoptosis-independent inhibition of cell migration and invasion

**DOI:** 10.1038/s41467-018-05204-6

**Published:** 2018-07-18

**Authors:** Anna Gorelick-Ashkenazi, Ron Weiss, Lena Sapozhnikov, Anat Florentin, Lama Tarayrah-Ibraheim, Dima Dweik, Keren Yacobi-Sharon, Eli Arama

**Affiliations:** 1 Department of Molecular Genetics, Weizmann Institute of Science, Rehovot, 76100 Israel; 2 Present Address: Department of Cellular Biology, University of Georgia, Athens, GA 30602-2607 USA

## Abstract

Maintenance of tissue integrity during development and homeostasis requires the precise coordination of several cell-based processes, including cell death. In animals, the majority of such cell death occurs by apoptosis, a process mediated by caspase proteases. To elucidate the role of caspases in tissue integrity, we investigated the behavior of *Drosophila* epithelial cells that are severely compromised for caspase activity. We show that these cells acquire migratory and invasive capacities, either within 1–2 days following irradiation or spontaneously during development. Importantly, low levels of effector caspase activity, which are far below the threshold required to induce apoptosis, can potently inhibit this process, as well as a distinct, developmental paradigm of primordial germ cell migration. These findings may have implications for radiation therapy in cancer treatment. Furthermore, given the presence of caspases throughout metazoa, our results could imply that preventing unwanted cell migration constitutes an ancient non-apoptotic function of these proteases.

## Abstract (web summary)

In addition to regulating programmed cell death, caspases also have non-apoptotic roles. Here, the authors show that low level caspase activity prevents cell migration to maintain tissue integrity.

## Introduction

Caspases are unique cysteine aspartate proteases mainly known for their crucial role in the execution of apoptotic cell death in metazoa^1–3^. Caspases are generally divided into initiators and effectors based on their structure and function in apoptosis. Initiator caspases are activated in distinct large multimeric protein complexes, whereas effector caspases are activated by the initiator caspases^4–6^. Activation of caspase-9, the initiator caspase of the intrinsic apoptotic pathway, is mediated by a heptameric, Apaf-1-based, adaptor complex known as the apoptosome^7^. Active caspase-9 then cleaves and activates effector caspases, such as caspase-3 and caspase-7, which in turn proteolytically digest hundreds of cellular substrates, culminating in cell death^8,9^. However, non-apoptotic roles of caspases, as well as caspase-independent alternative cell death pathways have also been described in metazoa^10,11^. Thus, caspases could have either evolved as dedicated metazoan-specific cell demolition enzymes or they could have originally carried out other functions unrelated to cell death^12,13^. Here, we describe a non-apoptotic role of caspases in maintaining epithelial tissue integrity in *Drosophila*. We show that epithelial cells can acquire mesenchymal cell-like fate, including loss of cell polarity, and gain of migratory and invasive capacities, either following ionizing irradiation or spontaneously during development. We also show that low levels of effector caspase activity potently inhibit this process, as well as another, distinct developmental cell migration process. These findings may extend our understanding of the role of caspases as tumor suppressors and have implications for radiation therapy in cancer treatment. Given that caspases are present throughout the animal kingdom, even among the most basal metazoa^14,15^, but are absent in unicellular organisms^13,16,17^, maintenance of tissue integrity by preventing unwanted cell migration, could be one of the most ancient non-apoptotic functions of this family of proteases.

## Results

### Ionizing irradiation triggers epithelial cell migration

**Fig. 1:**
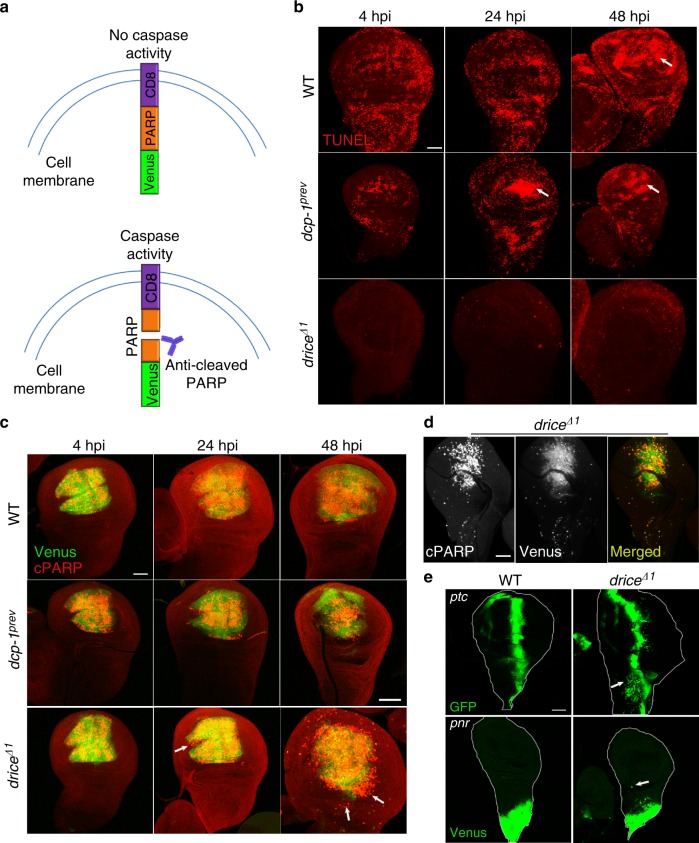
ICM is induced in *drice*^−/−^ but not *dcp-1*^−/−^ mutants. **a** A schematic representation of the CPV reporter of effector caspase activity. **b** Stereotypical clearance of TUNEL labeled cell corpses toward the pouch area (indicated by white arrows) in irradiated WDs. Note that the clearance occurs over more than 48 hpi in both WT and *dcp-1*^−/−^ WDs, and that apoptosis is essentially blocked throughout this period in the *drice*^−/−^ WDs. **c** Migrating cells in *drice*^−/−^ WDs display effector caspase (Dcp-1) activity. Effector caspase activity (i.e., CPV cleavage) is detected by an anti-cleaved PARP (cPARP) antibody (red). White arrows indicate cells that migrated away from their expression domain in the pouch area. **d** A *drice*^−/−^ WD at 48 hpi (50 Gy) showing that each migrating cell that displays caspase activity also displays Venus. **e** ICM is induced throughout the irradiated (50 Gy) WDs at 48 hpi. White arrows indicate either GFP- or CPV-expressing cells that migrated away from their expression domains at the anterior–posterior compartment boundary (*ptc*) or the notum region (*pnr*), respectively. Scale bars, 50 µm

We previously utilized the fruit fly *Drosophila* wing imaginal disc (WD), a relatively simple tissue mainly comprised of a monolayer of columnar epithelial cells, as a paradigm to investigate the apoptotic threshold of effector caspase activity following ionizing irradiation^18^. We used transgenic flies expressing CPV (CD8-PARP-Venus), a genetic reporter for effector caspase activity, which upon cleavage by Drice and Dcp-1, exposes a new PARP epitope that can be detected by an anti-cleaved PARP (cPARP) antibody (Fig. 1a). Using this reporter, we demonstrated that both Drice and Dcp-1, the *Drosophila* orthologs of caspase-3 and -7, become activated in irradiated WDs, and trigger apoptosis within 2.5-3 h post-irradiation (hpi). Functional genetic studies revealed that both caspases are activated to a similar extent and together account for all the detected effector caspase activity in the WDs, although Dcp-1 is far less efficient in triggering apoptosis than Drice in this context (albeit both caspases cleave CPV in a similar efficiency)^18^. Consistently, following a 50 Gy dose of γ-irradiation, dying cells were abundant in wild-type (WT) and *dcp-1*^*prev/prev*^ null mutant (*dcp-1*^−/−^) WDs, even 2 days after irradiation, whereas essentially no dying cells were detected in *drice*^*Δ1/Δ1*^ null mutant (*drice*^−/−^) WDs that only contain Dcp-1 effector caspase activity (Fig. 1b).

Taking advantage of this well-defined in vivo setup, we wanted to investigate the behavior of irradiated WD cells lacking the major effector caspase Drice. The *spalt (sal)*-Gal4 driver was used to obtain restricted expression of the CPV reporter in the pouch subdomain of the WD, a region corresponding to the adult wing, in the background of the *drice*^−/−^ and *dcp-1*^−/−^ mutants, and the WT control. Upon exposure of the larvae to a 50 Gy dose of γ-irradiation, caspase activity was observed in WDs from all three genotypes at 4, 24, and 48 hpi (Fig. 1c). Intriguingly, whereas in WT and *dcp-1*^−/−^ WDs this activity remained confined to the pouch (marked by the Venus fluorescence of the CPV reporter) at all the time points, *drice*^−/−^ WDs displayed many cPARP-positive cells outside of the reported expression domain that eventually scattered throughout the disc (Fig. 1c). Of note, each of the cPARP-positive migrating cells was necessarily also Venus-positive (Fig. 1d).

**Fig. 2:**
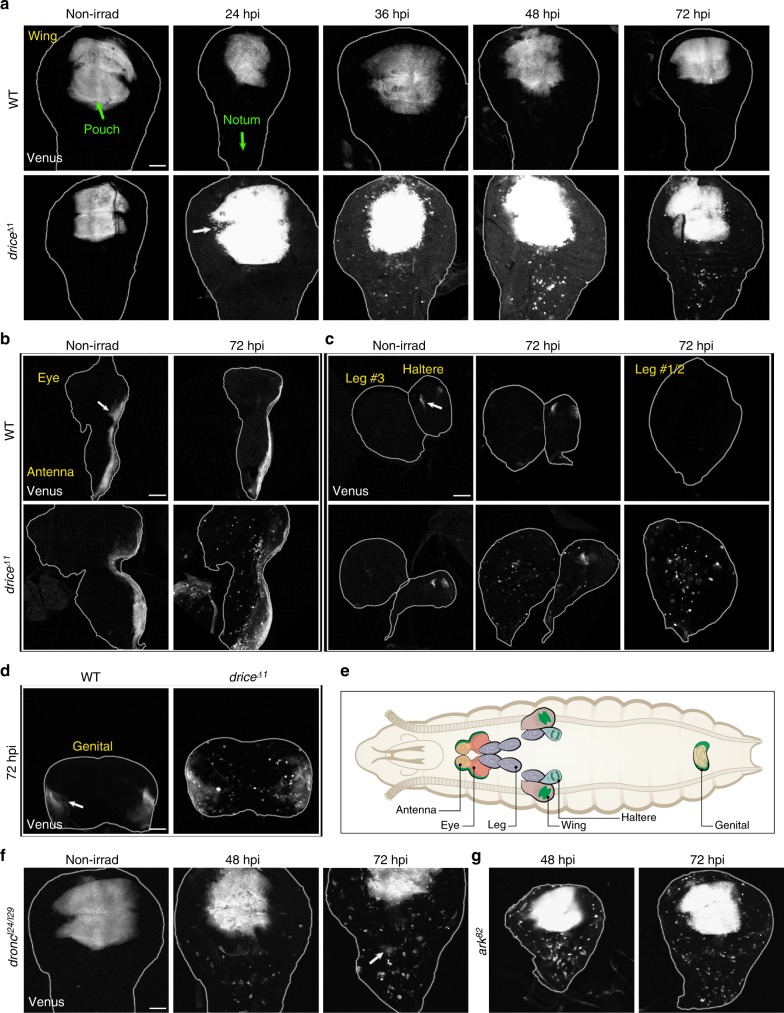
Irradiated epithelial cells compromised for caspase activity acquire migration capacity. **a** ICM is induced in irradiated (50 Gy) WDs where the major effector caspase Drice is completely inactivated, but not in irradiated WT WDs and non-irradiated WDs, starting at 24 hpi (white arrow) and peaking at 48 hpi. **b**–**d** ICM is induced in all the indicated irradiated (50 Gy) imaginal discs, including the leg discs (**c**) where *sal*-Gal4 is not expressed. Arrows indicate the *sal*-Gal4 expression regions. **e** Illustration of a *Drosophila* larva and the examined imaginal discs. The corresponding *sal*-Gal4 expression regions are indicated in green. **f**, **g** Inactivation of the apoptosome components can induce ICM. Irradiated *dronc*^*I24/I29*^ (**f**) and *ark*^*82*^ (**g**) mutant WDs (50 Gy) display multiple migrating cells, some of which are in clusters (arrow). Scale bars, 50 µm

To investigate this phenomenon, we first sought to establish a timeline for cell behavior in irradiated *drice*^−/−^ WDs, by monitoring the distribution of pouch cells over a set of increasing time points after irradiation (Fig. 2a). Venus-expressing cells could already be detected outside of the WD pouch by 24 hpi. The small number and close proximity of these cells to the pouch suggest that they originate in that region (white arrow in Fig. 2a). The size and spatial spread of this cell population increased at 36 hpi and peaked at 48 hpi, at which the Venus-expressing cells were detected as far as the distant notum subdomain of the WD, a region corresponding to the adult dorsal thorax (Fig. 2a). This temporal sequence implies that irradiated WD cells, in which effector caspase activity is strongly compromised, become motile and are capable of migrating over considerable distances. Notably, at 72 hpi, the number of remote Venus-expressing cells fluctuated (similar levels as at 48 hpi or reduced levels), either because these cells had migrated out of the WD, or because the WDs were too small at the time of irradiation (Fig. 2a). Henceforth, we will relate to this phenomenon as irradiation-induced cell migration (ICM).

### ICM is a cell autonomous process independent of phagocytosis

To negate the possibility that the motile “undead” cells might passively migrate within professional phagocytes, termed hemocytes^19,20^, we first monitored hemocyte distribution in the WDs of caspase mutant larvae following ICM. WDs from three transgenic fly lines expressing different hemocyte markers, *hemolectin (hml)*::GFP, *peroxidasine (pxn)*::GFP, and *hemese (he)*::DsRed, were analyzed at 48 hpi (Supplementary Fig. 1a). The hemocyte population, as detected with these markers, is far smaller than that of irradiation-induced migrating cells observed in these backgrounds (compare Supplementary Fig. 1a and Fig. 2a). Furthermore, in contrast to the readily apparent induction of migrating cells, hemocyte numbers rise minimally, if at all (Supplementary Fig. 1b). These findings were further genetically confirmed using null mutants for the *Drosophila* engulfment receptor Draper (the fly homolog of *C*. *elegans* CED-1), which is required for clearance by both professional (hemocytes) and non-professional phagocytes^21^. Indeed, the critical role of Draper in phagocytosis and clearance of dying cells was also demonstrated in both non-irradiated WDs, which displayed numerous uncleared developmentally dying cells (Supplementary Fig. 1c), as well as in irradiated WDs, in which the unique clearance pattern of dying cells toward the pouch area was completely abolished in the *draper* mutant (*drpr*^*Δ5*^; compare Fig. 1b and Supplementary Fig. 1c). Importantly, no effect on the pattern and levels of ICM was detected in WDs from *drice*^−/−^, *drpr*^−/−^ double mutants (Supplementary Fig. 1d). We conclude that ICM is a phagocyte-independent, cell motility phenomenon.

Our ICM model in WDs of *drice*^−/−^ mutant larvae was also confirmed by live imaging of primary WD cultures, demonstrating the cell autonomous nature of the migrating “undead” cells, which display dynamic protrusions (Supplementary Movie 1 and Movie 2).

### ICM is essentially induced in all of the imaginal disc cells

We next sought to determine whether the ICM phenomenon we observed for *drice*^−/−^ WD pouch cells is a general phenomenon. We first addressed this issue using the WD drivers *patched (ptc)*-Gal4 and *pannier (pnr)*-Gal4, to respectively express GFP or the CPV reporter either along the anterior–posterior compartment boundary (*ptc*) or in the notum region of the WD (*pnr*). Cells migrating out of their expression domains were clearly detected in both cases at 48 hpi, although the extent of migration from the *pnr*- and the *ptc*-expression regions was relatively restricted, presumably reflecting different migration potentials between cells in different WD regions (Fig. 1e).

We next made use of the *sal*-Gal4 driver to monitor ICM (at 48 and 72 hpi) in additional imaginal disc types (the deduced *sal*-Gal4 expression domains in the different types of imaginal discs are illustrated in green in Fig. 2e). Cells migrating out of the confined *sal*-Gal4 expression domains were readily detected in the eye-antenna, haltere, and genital imaginal discs (Fig. 2b-e and Supplementary Fig. 2). Moreover, although the *sal*-Gal4 driver is not expressed in the three leg imaginal disc pairs^22^, numerous Venus-positive cells could be detected throughout these structures, indicating that irradiation-induced migrating cells, which originated in other epithelial tissues, are highly invasive and capable of long-range migration (Fig. 2c, e and Supplementary Fig. 2).

**Fig. 3:**
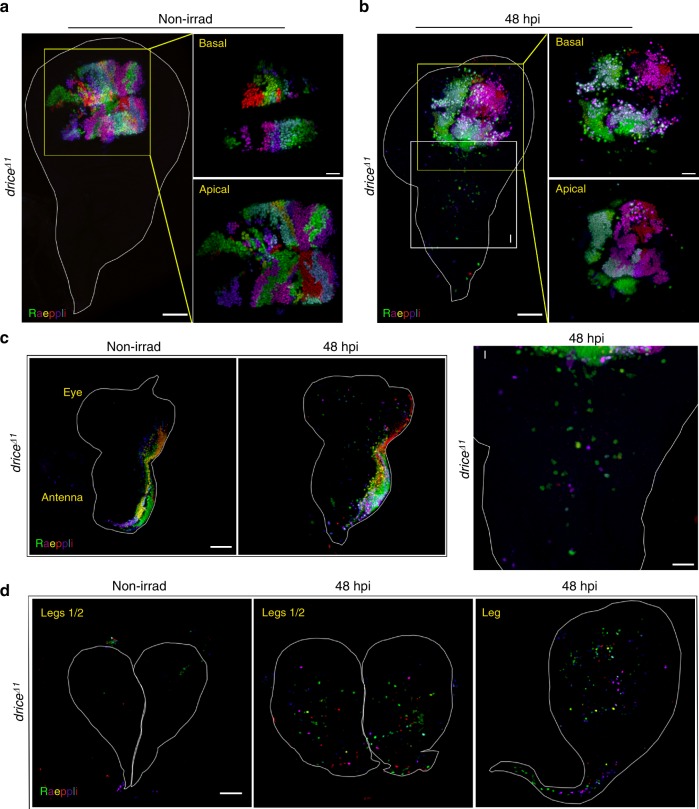
Migrating cells delaminate basally and display no directionality in cell migration. 3D projections of multiple fluorescent color clones generated by the Raeppli construct that was expressed under the *sal*-Gal4 driver in the background of the *drice*^−/−^ mutant. The imaginal discs were treated as indicated above each image, either non-irradiated (non-irrad) or irradiated (40 Gy, 48 hpi) to induce ICM in the wing (**a**, **b**), eye-antenna (**c**) and leg (**d**) imaginal discs. **a**, **b** Basal and apical optical sections of the areas marked by yellow squares were magnified and appear to the right. **a** A non-irradiated WD and the magnifications of the basal and apical optical sections of the pouch show regular arrangement of the tissue epithelia. **b** Irradiated WD and the magnifications of the basal and apical optical sections show massive basal delamination, albeit some migrating cells are also detected in the apical side. **b**, **I** Magnification of the area marked by the white square. Note that the different fluorescent clones are all represented in the migrating cells, indicating no specific directional migration. **c** Similar to the WD, ICM is also readily induced in the eye-antenna imaginal disc, showing no specific migratory directions and no clone (area) bias to drive migrating cells. **d** Invasion of migrating cells occurs in the irradiated leg imaginal discs following ICM induction. Note that the *sal-*Gal4 is not expressed in the leg discs. Scale bars (**a**–**d**), 50 µm, the magnifications in **a** and **b**, 20 µm

It is important to note that the endogenous expression of the Spalt protein is not required for ICM induction in the *sal*-Gal4 expression domain, as no correlation between migration efficiency and Spalt expression was detected in the migrating cells (Supplementary Fig. 3). In particular, both the long- and the short-distance migrating cells either expressed or did not express Spalt, even though they still expressed Venus, suggesting that the Venus signal does not reflect the perdurance and stability of the endogenous Spalt protein (Supplementary Fig. 3a). Endogenous Spalt was not detected in the cells that migrated to the leg imaginal discs, further suggesting that cell migration does not require Spalt expression (Supplementary Fig. 3b). Indeed, ICM could also be induced in the WD notum (marked by *pnr*-Gal4::CPV), an area which does not express either Spalt or *sal*-Gal4, further indicating that Spalt is not required for the induction of ICM (Fig. 1e and Supplementary Fig. 3c, II). Interestingly, notum area-originated migrating cells that made it to the pouch did not induce Spalt expression, implying that the migrating cells may not acquire the fate of the surrounding area (Supplementary Fig. 3c, I). Finally, since *sal*-Gal4 does not contain all the endogenous *spalt* regulatory sequences, its expression domain in the WD only partially overlaps with endogenous Spalt expression, driving wider expression in the pouch area and no expression in other WD areas (Supplementary Fig. 3a). Using the Raeppli tool (see the next paragraph), both endogenous Spalt negative and positive cells within the *sal*-Gal4 expression domain displayed similar tendencies to induce ICM (Fig. 3).

### ICM involves basal delamination and random migration paths

To be able to monitor the migratory paths of the irradiation-induced migrating cells and trace their subdomain origins, we used the *sal*-Gal4 driver to express a multicolor, lineage tracing, transgenic tool called Raeppli^23^. Clones of cells with multiple color combinations were induced in the *sal*-Gal4 expression domain in *drice*^−/−^ mutant wing and eye-antenna but not leg imaginal discs (Fig. 3a, c, d). Whereas essentially no migrating cells were detected in the non-irradiated imaginal discs, numerous migrating cells were observed in all the imaginal disc types at 48 hpi (Fig. 3). Examining basal and apical optical sections in the WD pouch revealed that the vast majority of the migrating cells delaminate basally (Fig. 3b). Tracing the subdomain origins of the migrating cells by color matching in the WDs, suggested no directionality in cell migration (Fig. 3b, I, c). Finally, the leg imaginal discs contained numerous migrating cells of various fluorescent colors, further demonstrating the long-distance migration capacity of these cells (Fig. 3d).

### ICM is inhibited by nonlethal levels of caspase activity

**Fig. 4:**
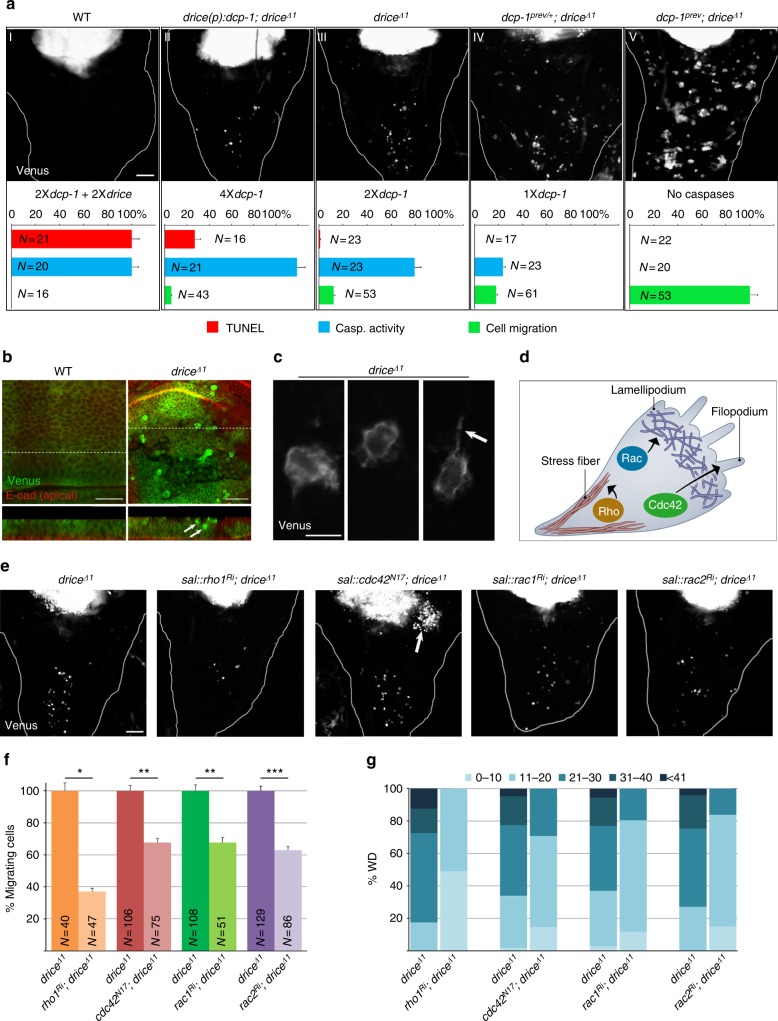
Effector caspase activity inhibits ICM independently of apoptosis. **a** Induction of ICM in WDs from a series of irradiated (18 Gy) transgenic and mutant flies with gradual decrease in their apoptotic potential. Each panel (**I–V**) contains the genotype, a representative WD with migrating cells at 48 hpi, the total number of *drice* and *dcp-1* gene copies, and three graph bars, indicating the apoptotic potential (TUNEL levels relative to WT, red), effector caspase activity levels (PARP [within the CPV] cleavage levels relative to WT, blue), and ICM levels (migrating cell levels relative to the *dcp-1*^−/−^; *drice*^−/−^ double mutant, green). *P* values were calculated as follows: for TUNEL, *P* < 0.001 between all the genotypes, one-way ANOVA followed by Tukey multiple comparisons post hoc test; for cPARP, *P* < 0.001 between all the genotypes except *P* < 0.05 between WT and *drice*^−/−^ and NS between WT and *drice:dcp-1*; *drice*^−/−^, one-way ANOVA followed by Tukey multiple comparisons post hoc test; for ICM levels, the significance between each of the different genotypes and the *dcp-1*^−/−^*; drice*^−/−^ double mutant was *P* < 0.001, two-way ANOVA followed by Tukey multiple comparisons post hoc test. Due to the variance in the double mutant, a similar analysis was then performed after excluding the double mutant to test the significant difference among the different genotypes (*P* < 0.001). Error bars indicate s.e.m. The examined WD numbers (*N*) are indicated for each graph bar. Scale bar, 50 µm. **b** WD pouch areas of the indicated genotypes after ICM induction (50 Gy, 72 hpi). The lower panels show *Z*-axis projections corresponding to the dashed lines regions in the upper panels. Arrows indicate compact and rounded delaminating/migrating cells that lost E-cadherin expression. Scale bars, 20 µm. **c** High magnification fluorescence images of three migrating cells in area proximal to the pouch. Arrow indicates a filopodium. Scale bar, 5 µm. **d** Illustration of a cell with the small GTPases that orchestrate cell movement. **e-g** Pouch area inactivation of the Rho small GTPases attenuates ICM. Representative images of irradiated WDs (18 Gy, 48 hpi) of the indicated genotypes (**e**), and the corresponding quantifications of ICM levels, presented either as migrating cell number averages relative to control (**f**) or as the percentage of WDs with each of five different migrating cell number categories (color coded by different shades of blue; **g**). Arrow indicates clustering of cells near the pouch. The examined WD numbers (*N*) are indicated for each graph bar. Error bars indicate s.e.m. *P < 0.05, **P < 0.01, ***P < 0.001, two-way ANOVA. Scale bar, 50 µm

Taken together, these observations suggest that imaginal disc epithelial cells become migratory upon irradiation, a process that is inhibited by effector caspase activity. We envisage two possible ways by which the effector caspases may inhibit ICM—either as an indirect consequence of their role as mediators of apoptosis, or more directly, through proteolysis of factors that promote cell migration. To assess which of these aspects of caspase function applies to ICM inhibition, we wanted to uncouple between caspase activity and apoptosis. For this, we generated a series of transgenic and mutant fly lines possessing decreasing levels of effector caspase activity, in order to reach a level of activity that is below the threshold required to induce apoptosis. We then quantified and compared apoptotic potential (the levels of dying cells detected by TUNEL labeling; 50 Gy γ-rays, 3-3.5 hpi), caspase proteolytic activity levels (CPV reporter cleavage levels; 50 Gy γ-rays, 3-3.5 hpi) and ICM levels (the combined area of the Venus fluorescent pixels proximal to the pouch; 18 Gy X-rays, 48 hpi) in WDs from larvae of the different lines (Fig. 4a). ICM was never induced in WT WD cells which displayed massive cell death as early as 3–4 hpi (Fig. 4a, I). However, following significant reduction of more than 70% in the apoptotic potential in flies expressing two transgenic copies of *dcp-1* under the *drice* regulatory regions, in the background of *drice*^−/−^ mutant, low levels of migrating cells were detected outside the WD pouch, indicating the generation of “undead” cells under these conditions (Fig. 4a, II). When the apoptotic potential was further decreased to about 1% of the WT level (in the *drice*^−/−^ mutants), ICM levels were doubled (Fig. 4a, III). Additional removal of a single gene copy of *dcp-1* (*dcp-1*^*−/+*^; *drice*^−/−^ mutants), further increased ICM levels and uncoupled between caspase activity and cell death, as cell death was completely abolished in the presence of the residual Dcp-1 activity (Fig. 4a, IV). Critically, these non-lethal levels of Dcp-1 activity could still potently inhibit ICM induction, since the removal of the last *dcp-1* gene copy (*dcp-1*^−/−^; *drice*^−/−^ double mutants) led to more than 5-fold increase in ICM levels (Fig. 4a, V). Collectively, these findings demonstrate that low levels of effector caspase activity, which are insufficient to trigger apoptosis, can potently inhibit ICM, thus uncovering an apoptosis-independent role of Dcp-1 in this process.

Similar results were also obtained following 40 Gy of X-irradiation, a dose equivalent to 50 Gy of γ-irradiation in the context of ICM induction (Supplementary Fig. 4a). Notably, the gradual increase in ICM levels does not involve proliferation of the migrating cells, as demonstrated both by staining with an anti-phosphohistone H3 (PH3) antibody and by EdU labeling, two cell division markers (Supplementary Fig. 4b, c).

The fact that not all the WD pouch cells migrate in the *drice*^−/−^ background at 48 hpi may suggest that other factors, besides reduced caspase activity, are also required for cell migration. Consistently, non-migrating cells are also detected in the *dcp-1*^−/−^; *drice*^−/−^ double mutants, which completely lack effector caspase activity, further indicating that the variability in the migration potential among different WD cell populations could not be solely attributed to inherent variations in the Dcp-1 activity levels. Indeed, when we examined the expression levels of activated Dcp-1 (cleaved Dcp-1; cDcp-1) in the short-distance and the long-distance migratory cells in the *drice*^−/−^ mutant WDs, no correlation was detected between cDcp-1 levels and the migratory distances covered by the cells (Supplementary Fig. 5). Therefore, once caspase activity is reduced below the apoptosis induction threshold, the ICM induction potential may still vary between different cells, presumably due to variations in expression levels of pro-migratory factors.

### ICM is reminiscent of the epithelial–mesenchymal transition

Our initial observations suggested that the shape of the migrating cells during ICM was morphologically distinct from that of the columnar cells in the original WD epithelium layer. We therefore closely examined the morphology of the migrating cells as they originate in the WD pouch area of irradiated *drice*^−/−^ mutants at 72 hpi. The WDs were also stained to visualize E-cadherin, a key, apically localized adherens-junction resident protein, which connects between neighboring cells^24^. Analysis of confocal optical sections and *Z*-axis projections of WDs following ICM induction revealed that the delaminating/migrating cells lose apicobasal polarity, as seen by the loss of E-cadherin expression, and undergo dramatic morphological changes, during which they acquire a highly compact and rounded shape, and often contain extended cellular projections reminiscent of lamellipodia (Fig. 4b, c; Supplementary Movie 2). Interestingly, ICM-associated cell morphology, molecular and behavioral changes are reminiscent of a physiological process known as epithelial–mesenchymal transition (EMT), during which polarized epithelial cells undergo multiple biochemical changes, culminating in enhanced migratory and invasive capacities^25,26^.

The acquisition of enhanced migratory and invasive capacities suggests the involvement of extensive cytoskeletal dynamics. The Rho family of small guanosine triphosphate (GTP)-binding proteins (GTPases) are important regulators of actin and adhesion organization, and facilitate cell motility by controlling the formation of lamellipodia and filopodia protrusions (Fig. 4d)^27^. Interestingly, data mining for identification of caspase substrates in publicly available proteomics data sources identified at least four major Rho GTPases, in which the caspase cleavage sites are highly conserved between flies and mammals^28–30^. To test for possible involvement of this canonical signaling pathway in ICM, we inactivated these four Rho GTPases in the pouch area of WDs from irradiated *drice*^−/−^ mutants (Fig. 4e). Specific pouch epithelial cell knockdown of *rho1*, *rac1*, and *rac2* using RNA interference (RNAi), and overexpression of a dominant-negative form of Cdc42, all significantly reduced the number of migrating cells as compared to the control (Fig. 4e–g). Notably, when Cdc42 was inactivated, the WDs often exhibited large clusters of migrating cells near the pouch area, which could be attributed to some of the non-overlapping functions of these Rho GTPases (Fig. 4d, e). These findings further demonstrate the cell autonomous nature of the migration capacity during ICM.

**Fig. 5:**
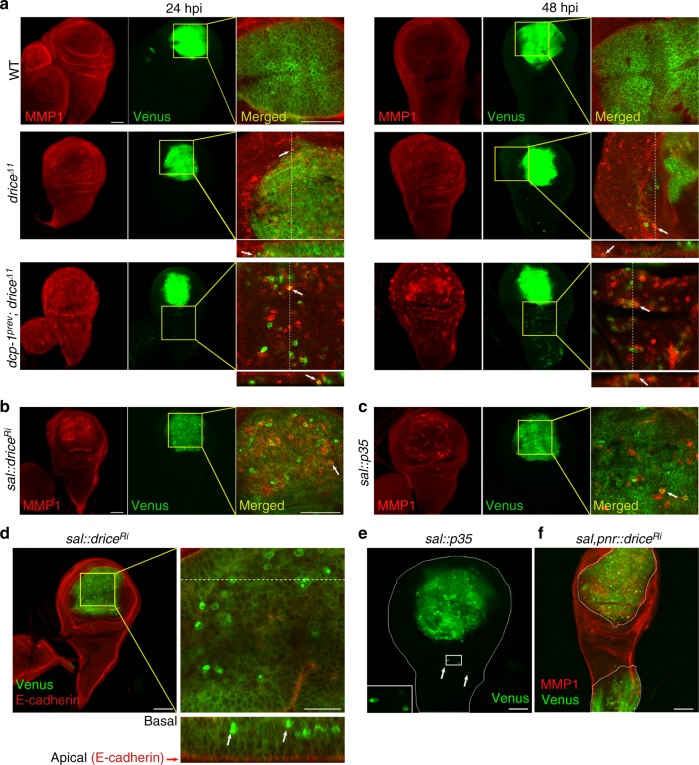
Compromised caspase activity in the invaded tissue enhances cell invasion. Migrating cells are marked in green (intense Venus expression). **a** ICM levels directly correlate with MMP1 expression levels. Selected images of WDs from the indicated genotypes at 24 and 48 hpi (50 Gy) stained with anti-MMP1 antibody (red). The images on the right of each panel represent higher magnifications of the merged (red and green) channel areas indicated by the yellow squares. Dashed lines indicate the regions for which *Z*-axis projections (bottom) were generated. **b**, **c** Confined knockdown of *drice* or overexpression of p35 in the pouch areas of irradiated (40 Gy or 50 Gy, respectively, 48 hpi) WDs induces MMP1 expression only in the pouch. Images were organized as in **a**. White arrows in **a**–**c** indicate migrating cells that are also MMP1-positive, as this feature appears to be transient. **d** Knockdown of *drice* in the pouch area induces migrating cells that are mainly confined to this area of the irradiated (50 Gy, 48 hpi) WD. Scale bar, 50 µm. The image on the right is a higher magnification of the area indicated by the yellow square; scale bar, 20 µm. White arrows indicate migrating cells displaying delamination pattern, loss of apical E-cadherin expression, and cell morphology, which are highly reminiscent of the migrating cells induced when effector caspase function is compromised in the entire larva (compare with Fig. 4b). A dashed line indicates the region for which a *Z*-axis projection (bottom) was generated. **e** Pouch area overexpression of p35 also induces area-confined ICM in irradiated (40 Gy, 48 hpi) WD. The inset contains a higher magnification image of the area marked by a rectangle. White arrows indicate relatively rare migrating cells which invaded the surrounding WT tissue. **f** Simultaneous knockdown of *drice* in the pouch and the notum induces area-confined ICM and MMP1 expression in irradiated (40 Gy, 48 hpi) WD. Scale bars in **a–c**, **e**, **f**, 50 µm

Local changes in tissue integrity are often associated with the remodeling of the extracellular matrix (ECM). Matrix metalloproteinases (MMPs) constitute a major family of ECM endopeptidases involved, amongst other functions, in ECM remodeling, EMT and tumor cell invasion^31–33^. We thus tested for possible association between MMP expression and caspase activation during ICM, using an antibody against MMP1, one of two *Drosophila* MMPs, involved in basement membrane degradation and tumor invasion^34^. Interestingly, we uncovered an inverse correlation between effector caspase activity and MMP1 expression following irradiation (Fig. 5a). No or minimal MMP1 expression was observed in WT and caspase mutant WDs at 0 and 4 hpi, respectively. However, MMP1 expression became prominent throughout the WD in the *drice*^−/−^ single mutant and the *dcp-1*^−/−^; *drice*^−/−^ double mutants, but not in WT discs, at 24 and 48 hpi (Fig. 5a). Notably, similar to the gradual elevation of ICM levels, MMP1 expression also increased with time and with the gradual decrease in effector caspase activity, peaking in the *dcp-1*^−/−^; *drice*^−/−^ double mutant at 48 hpi (Fig. 5a). This trend was also observed following both restricted knockdown of *drice* or overexpression of the baculovirus effector caspase inhibitor protein p35 (which inhibits both Drice and Dcp-1) in the pouch area, where, similar to ICM induction, MMP1 expression was confined to the affected (pouch) area (Fig. 5b, c). It is noteworthy that despite the general inverse correlation between caspase activity levels and MMP1 expression in the WDs following irradiation, not all of the migrating cells expressed MMP1 (Fig. 5a–c). This may indicate that secreted MMP1 is only required transiently during the transition to migrating cells. Moreover, knockdown of *mmp1* and *mmp2* in the pouch area did not attenuate ICM in the *drice*^−/−^ mutant WDs, suggesting that the function of the two MMPs could be redundant and/or that other ECM proteinases might also be involved in the ECM remodeling during ICM (Supplementary Fig. 6d-f). Nevertheless, the accumulation of MMP1 in the caspase-compromised tissue may reflect extensive ECM remodeling, which could account for the high sensitivity of this mutant tissue to be invaded by the migrating cells.

Since MMP1 was reported as one of the downstream targets of the JNK signaling^35^, we examined the expression of active JNK (phosphorylated JNK; pJNK) during ICM induction. Similar to the increased MMP1 expression in the *drice*^−/−^ mutant and *drice* knockdown WDs, increased pJNK expression was also detected in these mutant WDs. Unexpectedly however, while MMP1 expression further increased in the *dcp-1*^−/−^; *drice*^−/−^ double mutant, pJNK expression significantly decreased in the double mutant WDs, suggesting that at least in this process, MMP1 expression is induced independently of JNK signaling (Supplementary Fig. 6b; also compare to Fig. 5a). This idea is also supported by genetic experiments, as knockdown of the *jnk* gene (*bsk*^*Ri*^) and overexpression of the JNK inhibitor Puckered (Puc), both failed to attenuate ICM induction (Supplementary Fig. 6c, f).

### Caspase activity in the invaded tissue affects ICM potency

An important aspect in characterizing ICM is the degree to which the invaded tissue also influences the behavior of the migrating cells. To address this question, we generated flies in which caspase activity was compromised only in a restricted WD region, by using *sal*-Gal4 to drive RNAi against *drice* or overexpress p35 in the pouch of irradiated larvae. Pouch-derived cells were visualized by virtue of the Venus fluorescence of the co-expressed CPV reporter. Unexpectedly, although many cells that display the ICM hallmarks described above could be visualized at 48 hpi, they were mainly confined to the pouch area, and only rarely appeared in the surrounding WT tissue (Fig. 5d, e). Similar results were obtained when *drice* was simultaneously knocked down in two distinct and separate WD subdomains, the pouch and notum, displaying migrating cells within the two affected subdomains, while the bridging WT tissue was devoid of these cells (Fig. 5f). The ability to efficiently invade the surrounding tissue, observed when effector caspase function is compromised in the entire larva, appears therefore to require a non-cell autonomous factor/condition present in the invaded tissue, which is controlled by caspase activity. Along these lines, the detection of the inverse correlation between MMP1 expression and caspase activity, following ICM induction in the caspase mutant WDs, suggested that at least in part, ECM remodeling could render the caspase-compromised tissue more prone for cell invasion (Fig. 5a). Indeed, when caspase activity was only compromised in specific WD domains, MMP1 expression was significantly elevated only in the mutant areas but not in the surrounding WT tissue (Fig. 5b, c, f).

Of note, ECM remodeling is not the only difference between the WT and caspase mutant tissues, as following irradiation, the surrounding WT tissue contains lethal levels of caspase activity, leading to massive apoptosis and accumulation of cell corpses. Therefore, the reduced invasion efficiency of the migrating cells to the surrounding WT tissue could also be attributed to physical barriers/obstacles generated by the overwhelming numbers of persistent cell corpses in the WT tissue (see also Fig. 1b). Consistently, similar to the irradiation-induced migrating cells that delaminate basally, apoptotic cells are also detached from the tissue and move towards the basal lamina^36^.

### ICM is triggered by the DNA damage response pathway

We next wanted to uncover the signaling pathway that induces ICM following irradiation and identify its point of bifurcation from the signal that triggers caspase activation and apoptosis. Previously, another phenomenon called apoptosis-induced proliferation (AiP), in which dying cells secrete mitogenic signals that promote excessive cell proliferation in the surrounding WT tissue, was revealed in the fly imaginal discs by the induction of “undead” cells (i.e., cells induced to undergo apoptosis but at the same time also compromised for caspases)^37^. However, the signals that induce AiP and ICM appear to be distinct. Whereas AiP requires the apoptosome components Dronc and Ark (orthologs of caspase-9 and Apaf-1, respectively), ICM was induced, rather than attenuated, in WDs from *dronc* and *ark* null mutants (Fig. 2f, g). Furthermore, AiP can be induced at the level of the core apoptotic machinery, e.g., by the co-expression of p35 and a *Drosophila* Inhibitor of apoptosis protein 1 (Diap1) antagonist (e.g., Reaper or Hid), whereas pouch area knockdown of *diap1* failed to induce ICM in the *dcp-1*^−/−^; *drice*^−/−^ double mutant WDs (Supplementary Fig. 7a).

**Fig. 6:**
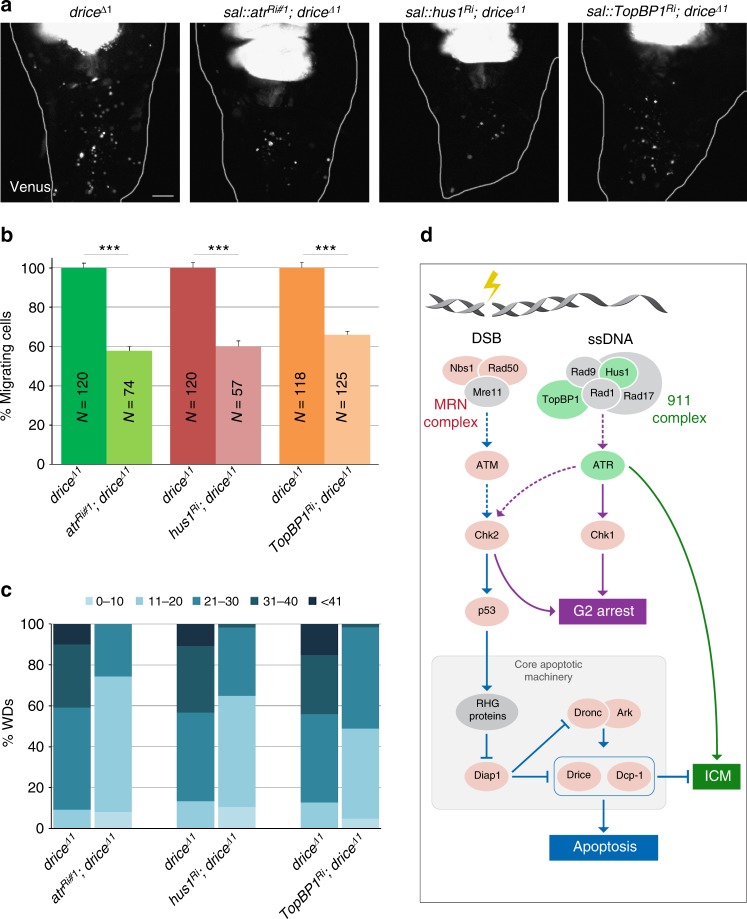
The 9-1-1/ATR branch of the DNA damage response pathway mediates ICM induction. **a–c** Pouch area knockdown of components in the 9-1-1/ATR signaling pathway attenuates ICM. Representative images of irradiated WDs (18 Gy, 48 hpi) of the indicated genotypes (**a**), and the corresponding quantifications of ICM levels, presented either as migrating cell number averages relative to control (**b**) or as the percentage of WDs with each of five different migrating cell number categories (color coded by different shades of blue; **c**). The examined WD numbers (*N*) are indicated for each graph bar. Error bars indicate s.e.m. ***P < 0.001, two-way ANOVA. Scale bar, 50 µm. **d** Illustration of the two branches of the DNA damage response pathway induced by irradiation. Tested components for effects on ICM induction are indicated in red (for no effect) and green (for having an effect). Untested/unconfirmed components are in gray. Dashed lines indicate suggested interactions based on data from mammalian systems or when direct regulation was not observed in *Drosophila*

**Fig. 7:**
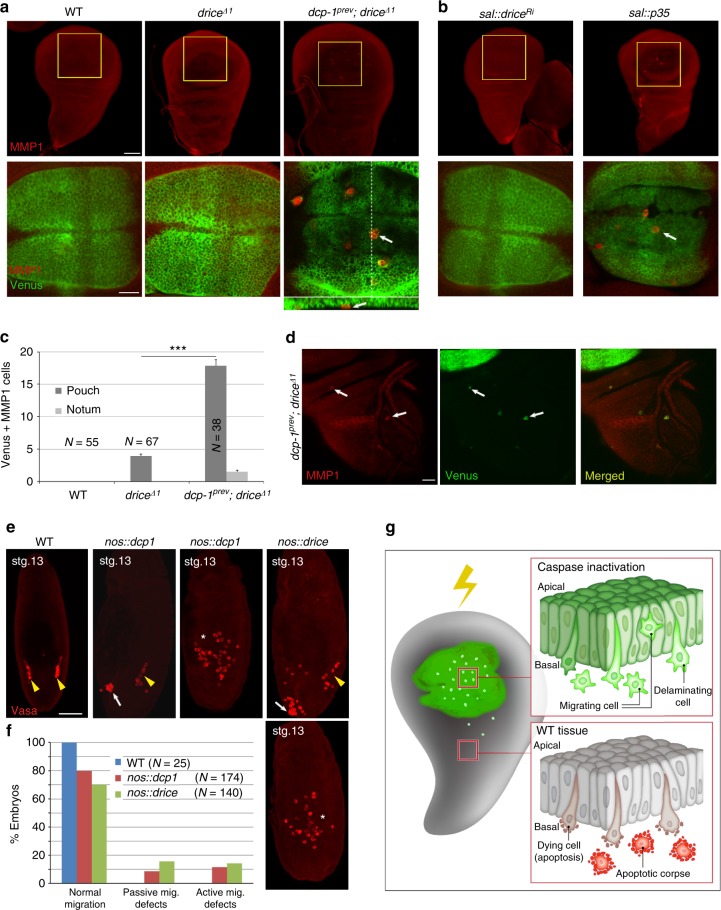
Nonlethal caspase levels inhibit spontaneous emergence of migrating cells and PGC migration. **a**, **b** Delaminating/migrating cells spontaneously emerge in non-irradiated WDs (of the indicated genotypes) compromised for caspase activity. The pouch areas in the lower row (marked in green by the *sal*::CPV) are the corresponding magnifications of the WD regions marked in yellow squares in the upper row. Arrows indicate delaminating/migrating cells detected by virtue of their compact shape (intense Venus fluorescence; green) and MMP1 expression (red). The *Z*-axis projection in **a** corresponds to the dashed line region in the upper image. Scale bars, upper row 50 µm and lower row 20 µm. **c** Quantifications of the intense Venus and MMP1 double-positive cells in **a**. “Pouch” and “Notum” indicate the WD areas in which the cells were detected. The examined WD numbers (*N*) are indicated for each graph bar. Error bars indicate s.e.m. ***P < 0.001, two-tailed unpaired Student’s t test. **d** Some of the intense Venus and MMP1 double-positive cells in the non-irradiated *dcp-1*^−/−^; *drice*^−/−^ double mutant WDs migrated away from the pouch (arrows; quantified in **c**). Scale bar, 20 µm. **e** The developmental migration of PGCs is affected by low increase in effector caspase levels. Stage 13 embryos overexpressing the full length *dcp-1* and *drice* coding regions (zymogens) in PGCs using the *nos*-Gal4 driver (which drives both maternal and zygotic expressions). PGCs are marked by anti-Vasa antibody staining. Yellow arrowheads indicate PGCs that correctly migrated to the gonads. White arrows and asterisks indicate PGCs that failed to migrate properly in the first and second migration phases, respectively. **f** Quantification of the percentage of stage 13 embryos with migration defects in genotypes shown in **e**. The numbers of examined embryos (*N*) are indicated. Scale bar, 50μm. **g** A model of irradiation-induced cell migration and apoptosis in the WD epithelial tissue. The green area (pouch) indicates a subdomain that is severely compromised for effector caspase activity, whereas the gray areas indicate WT tissue

Our observations suggest that irradiation activates an EMT-like inducing pathway in imaginal disc epithelial cells, which is caspase-sensitive. To gain insight into the molecular nature of this pathway, we performed an RNAi-based candidate screen for signaling pathway components which, upon knockdown, attenuate ICM induced in the *drice*^−/−^ mutant WDs. Significantly, pouch-specific knockdown of components in one of the branches of the DNA damage response pathway^38,39^, including the ATM-related kinase ATR, the 9-1-1 complex component Hus1 and the ATR activator TopBP1, all attenuated ICM levels by about 40% (Fig. 6; Supplementary Fig. 7c, d, f). Note that the specificities of the *atr* and *chk1* RNAi lines were tested in a functional assay in situ (Supplementary Fig. 7e). In contrast, knockdown of *chk1*, the best studied ATR effector in the DNA damage checkpoint^40^, failed to attenuate ICM (Supplementary Fig. 7c-f). Furthermore, knockdown of several components in the parallel DNA damage response pathway which triggers apoptosis^39^, including Chk2, ATM and components of the MRN complex, also failed to affect ICM (Fig. 6d; Supplementary Fig. 7f). Importantly, flies homozygous for a null allele of the mammalian p53 homolog, *dp53* (*p53*^*5A–1–4*^), a major mediator of irradiation-induced apoptosis in *Drosophila*^41^ (Fig. 6d), displayed no ICM attenuation in the background of *drice*^−/−^ mutant (Supplementary Fig. 7b). Of note, inactivation of p53 in an otherwise WT background could not trigger ICM (at 48 hpi), likely due to the p53-independent apoptosis that occurs at 18 hpi^42^, a time when ICM is still undetected. We conclude that, following irradiation, the two branches of the DNA damage response pathway are activated and trigger distinct responses with different kinetics: a rapid response (2.5-4 hpi)^18^ mediated by p53 and likely the upstream MRN/ATM/Chk2 pathway that triggers apoptosis, and a significantly slower response (24-48 hpi) mediated by the 9-1-1/ATR pathway, which induces ICM (Fig. 6d; Fig. 7g).

### Spontaneous emergence of migrating cells in caspase mutants

The findings that irradiation promotes an EMT-like process that is potently inhibited by low levels of effector caspase activity, far below the threshold required to induce apoptosis, may point to a physiological, non-apoptotic role for effector caspases in maintaining tissue integrity. To address this issue, WDs from non-irradiated *drice*^−/−^ mutants and *dcp-1*^−/−^; *drice*^−/−^ double mutants, expressing the CPV reporter in the pouch area, were stained to visualize MMP1-positive delaminating/migrating cells. Importantly, whereas WT WDs never contained such cells, spontaneously appearing delaminating/migrating cells were detected in WDs from the *drice*^−/−^ mutant (Supplementary Fig. 8a; Fig. 7c). Migrating cell numbers increased significantly (4 fold) in the *dcp-1*^−/−^; *drice*^−/−^ double mutant (Fig. 7a, c), which sometimes also contained cells that migrated away from the pouch toward the notum (Fig. 7c, d). Likewise, migrating cells were readily detected following overexpression of p35 in the pouch (Fig. 7b). Note that these numbers are underestimations, as to be rigorous, only the CPV and the transiently expressed MMP1 double-positive cells were counted. Thus, similar to ICM, this phenomenon is inversely correlated to the levels of effector caspase activity, displaying the highest levels of migration and invasion potencies when the caspases are completely abolished (Fig. 7c, d).

### Nonlethal caspase levels attenuate PGC migration

Previous work by Montell and colleagues suggested an apoptosis-independent role for Dronc and Ark in border cells migration (BCM) during *Drosophila* oogenesis^43^. However, BCM represents a different type of migration than ICM, and both may be controlled by distinct mechanisms. As opposed to ICM where cells migrate individually, BCM is a paradigm of collective migration where cells are attached to one another and move in a group. Furthermore, Dronc inhibits BCM independently of the effector caspases, which may suggest a non-catalytic role of Dronc in this process^43,44^. To examine whether the inhibitory effect of effector caspases on cell migration operates in additional developmental contexts, we turned to primordial germ cell (PGC) migration during *Drosophila* embryogenesis. In this process, PGCs migrate in two steps: first as part of global tissue movement during stages 7–9 (“passive”), and then during stages 10–13, they lose adhesion to each other and individually migrate through the posterior midgut (“active”)^45^. Since the abundance of pro-caspases is directly proportional to caspase activity level^18^, we used the *nanos (nos)*-Gal4 driver to overexpress the *dcp-1* and *drice* zymogens both maternally and zygotically in PGCs, in order to increase the basal level of effector caspase activity in these cells, yet keep the activity below the threshold required to induce apoptosis (Supplementary Fig. 8b, c). Significantly, migration defects were observed in 20% and 30% of the *dcp-1* and *drice* overexpressing PGCs, respectively (Fig. 7e, f). Of note, these flaws in PGC migration occurred during both phases of migration, as indicated by the patterns of the PGCs at stage 13, which may imply that the PGCs acquire autonomous migration capacities already during the first migration phase (Fig. 7e). Taken together, these findings demonstrate the ability of the effector caspases to potently inhibit cell migration with activity levels that are far below the thresholds required to trigger apoptosis.

## Discussion

The studies described here, as well as the recent demonstrations of the widespread presence of nonlethal caspase activity in *Drosophila* tissues^46,47^, raise the possibility that maintenance of tissue integrity and prevention of unwanted cell migration and invasion might be a more general property of the effector caspases at their basal activity levels. Intriguingly, caspases appear to have evolved together with the transition to multicellularity in metazoa^48,49^. Given the necessity to stabilize the multicellular state^50^, it is reasonable to expect that efficient mechanisms have evolved in order to maintain tissue integrity and generate cellular barriers that partition spaces and functions. Indeed, reverse evolution to unicellularity in non-metazoans was also noted^49,51^, as well as the idea that cancer cells may reactivate dormant pre-existing unicellular functions^52–54^. Although additional work in other metazoan organisms besides *Drosophila* is required in order to generalize our findings, it is still attractive to propose that the evolvement of caspases could have been part of these mechanisms.

Phenomena during which epithelial cells (some of which were locally compromised for caspase activity) acquire short-distance/restricted delamination and/or migration capacity have been previously observed in *Drosophila*^55–58^. However, none of these phenomena displayed the dramatic ICM potency and long-distance migration and invasion capacity, indicating that ICM is a distinct phenomenon (see also the Supplementary Discussion).

Our discovery that ionizing irradiation promotes acquisition of cell migration and invasion capacities when caspase activity is compromised is not only of an academic interest, as radiotherapy has been a common practice in cancer treatment, and pro-metastatic effects^59^ should be taken into consideration, in particular since cancer cells develop resistance to apoptosis^60^. In addition, our results that compromised caspase activity in the invaded tissues significantly increased the invasion efficiency of the migrating cells during ICM, could imply “good” prognosis to patients with de novo tumors. However, “poor” prognosis might be applied in patients with a genetic predisposition to compromised caspase activity. Future equivalent studies in mammals will illuminate whether and to what extent the ICM phenomenon can be applied to cancer patients.

## Methods

### Fly strains

Flies were grown at 25 °C. WT controls correspond to the genetic background of each experiment as follows: *sal-*Gal4*/UAS*-CPV (Fig. 1c, Fig. 2, Fig. 4, Fig. 5a, Fig. 7a–d, Supplementary Fig. 4, Supplementary Fig. 6a), *nos*-Gal4 (Fig. 7e, f), *UAS*-CPV (Fig. 1b), *ptc*-Gal4; *CD8*-GFP and *pnr*-Gal4, *UAS*-CPV (Fig. 1e), *yw* (Supplementary Fig. 7e), *GMR*-Gal4 (Supplementary Fig. 8c), *sal*-Gal4 (Supplementary Fig. 1c). Mutant and transgenic fly lines used in this study are as follows: *sal-*Gal4 driver (Konrad Basler, University of Zurich, Zurich, Switzerland), *Ptc-*Gal4 and *GMR-*Gal4 (lab stocks), *pnr-*Gal4 (Bloomington Stock #3039), *UAS*-CPV^61^, *UAS*-puc^62^, *dronc*^*I24*^ and *dronc*^*I29*^^63^, *drice*^*∆1*^^64^, *dcp-1*^*prev*^^65^, *ark*^*82*,^^66^, *p53*^*5A-1-4*^,^67^, *drpr*^*∆5*^ (^21^; the lethality and sterility were removed from the background by Estee Kurant, University of Haifa, Israel, and Kim McCall, Boston University, USA), *drice(p):dcp-1*^18^, *UAS*-p35 and *UAS*-hid7 (Hermann Steller, Rockefeller University, USA), *UAS*-CD8-GFP (Oren Schuldiner, Weizmann Institute, Israel), *hml-*Gal4*, UAS*-eGFP and *he-*Gal4, *UAS*-DsRed (Joe Rodriguez, Rockefeller University, USA), *pxn*-Gal4, *UAS*-GFP (Amir Orian, Technion, Israel), *cdc42*^*N17*^^68^, *nos*-Gal4 (Mark Van Doren, Johns Hopkins University, USA), *UAS*-fl-Dcp-1 (Kim McCall, Boston University, USA), *UAS*-drice-venus (Graeme Davis, UCSF, USA).

RNAi lines against the following genes were obtained from the Vienna *Drosophila* RNAi Center (VDRC): *drice* (GD28065), *rho1* (GD12734), *rac1* (GD49246), *rac2* (GD28926), *atr* #1 (GD11251), *hus1* (GD15881), *TopBP1* (GD31431), *chk1* (KK110076), *rad50* (KK103394), *nbs1* (KK110366), *atm* (KK108074), *chk2* (KK110342), *mmp1* (KK101505), *mmp2* (KK107888) and *bsk* (KK104569). Additional RNAi lines against *Diap1* (Pascal Meier, ICR London, UK) and *atr* #2 (the TRiP collection at Harvard Medical School; Bloomington stock #41934).

Stocks used for the Raeppli experiments are *hs*-FLP (Bloomington stock #8862) and Raeppli-NLS^23^(Bloomington stock #55086).

### Immunofluorescence staining and visualization

Irradiation was given to tubes and bottles containing larvae. Imaginal discs were dissected from third instar larvae either before or at different time points after treatment with ionizing radiation (for 48 hpi we allowed ± 2 h). Essentially two doses of irradiation were used in this study, high (50 Gy γ-rays or 40 Gy X-rays) and low (18 Gy X-rays), using either the Millenium 870 (Mainance, UK) for γ-irradiation or the XRAD 320 (Precision X-ray, USA) for X-irradiation.

Genotype order of imaginal disc dissections was randomized for each experiment.

To visualize the migrating cells, imaginal discs were fixed for 20 min at room temperature (RT) in 4% paraformaldehyde (PFA) in PBS, washed three times in PBS for 10 min, mounted and directly examined under a confocal microscope to visualize the Venus, GFP or the multiple Raeppli fluorescence.

Antibody labeling of the WDs was performed on the fixed samples, which were washed three times (3 × 10 min) in PBX (PBS with 0.1% TritonX-100), blocked in PBS/BSA (1% BSA in PBS) for 1 h at RT, and incubated overnight (ON) with the primary antibody (diluted in 1% PBS/BSA) at 4 °C. The samples were then washed in PBX, incubated with the secondary antibody for 90 min at RT, washed again in PBX and mounted.

For embryo staining, embryos were collected on *Drosophila* fruit juice egg plates with a dollop of thick yeast paste and were aged to the corresponding stages. After aging, the embryos were collected in mesh baskets, dechorionated with 50% bleach, fixed for 20 min (4% formaldehyde in PEMS [0.1 M Pipes pH = 6.9, 2 mM MgSO4, 1 mM EGTA]), devitellinized using heptane/methanol, and stained using standard protocols.

Images were taken on a confocal microscope (Zeiss LSM510, LSM710, LSM780, and LSM800).

### Antibodies

The primary antibodies used in this study were rabbit polyclonal anti-cleaved human PARP (1:500, Ab2317; Abcam), rabbit polyclonal anti-PH3 (1:200, 06-570; Millipore), rabbit anti-Vasa (1:250, sc-30210; Santa Cruz), rabbit anti-Spalt (1:1000, Adi Salzberg, Technion, Israel), rabbit anti-cleaved Dcp-1 (1:75, 9578; Cell Signaling), mouse anti-phospho SAPK/JNK (1:100, G9, 9255; Cell Signaling) and the following monoclonal antibodies from the Hybridoma Bank (DSHB): mouse anti-MMP1 hemopexine domain (1:10, 14A3D2 supernatant; Fig. 5a–c, Fig. 7a–d and Supplementary Fig. 6d) and catalytic domain (1:50, 5H7B11 concentrate; Fig. 5f), rat anti-E-cadherin (1:50, DCAD2 concentrate). Secondary antibodies were obtained from Jackson Immuno-Research (1:250).

### TUNEL labeling

WDs were fixed in 4% PFA for 20 min, washed twice in PBS (2 × 5 min), washed twice (2 × 10 min) in 1xBSS (5xBSS: 270 mM NaCl, 200 mM KCl, 37 mM MgSO_4_, 12 mM CaCl_2_.2H_2_O, 24 mM tricine, 1.8% glucose, and 8.5% sucrose), followed by two washes (2 × 5 min) in PBTw (0.1% Tween 20 in PBS). The samples were then refixed in 4% PFA for 20 min, washed five times (5 × 5 min) in PBTw, incubated in equilibration buffer (ApopTag kit; Millipore) for 1 h, followed by ON incubation with the TdT enzyme in reaction buffer (ratio 3:7; ApopTag kit) at 37 °C. The reaction was stopped by replacing the reaction mix with stop buffer (diluted 1:34 in dH2O; ApopTag kit) and incubation for 3-4 h at 37 °C. The samples were then washed three times (3 × 5 min) in PBTw, blocked in BTN solution (1xBSS, 0.3% Triton X-100, and 5% normal goat serum) for 1 h at RT, and incubated ON in the dark with anti-digoxigenin antibody solution (diluted 47:53 in blocking solution; ApopTag kit) at 4 °C. Samples were then washed four times (4 × 20 min) in 1xBSS, and mounted in Fluoromount-G (SouthernBiotech).

TUNEL labeling of embryos was performed following a modified manufacturer’s protocol^69^. To induce apoptosis in germ cells we used embryos laid by *nos*-Gal4 females mated to *UAS*-hid males.

### EdU labeling

Dissected WDs were quickly transferred to PBS on ice for the duration of the dissection. The PBS solution was then replaced with EdU solution (10 µM [Click-iT^®^ Plus EdU Imaging Kits, Molecular Probes] in standard Schneider’s medium [Biological Industries]) and incubated for 30 min at RT. WDs were rinsed with PBS and fixed in 4% PFA in PBS for 20 min at RT. The samples were then rinsed in 3% BSA in PBS, washed again (2 × 10 min), and then incubated in 0.1% Triton X-100 in PBS for 20 min at RT. The following steps were performed according to the kit protocol. The washes were done in 3% BSA in PBS for 10 min each wash.

### Live imaging of migrating cells

WDs were dissected in standard Schneider’s medium (Biological Industries) and transferred to 150 µm Schneider’s medium in an eight-well chamber (ibidi µ-slide; 80826) coated with Matrigel (BD Bioscience, diluted 1:10 in PBS, and left for 30 min at 37 °C). The WDs were allowed to adhere to the chamber for 30 min at RT, and then were gently covered with manually size adjusted cover slip. Visualization was made on a confocal microscope. For Supplementary Movie 1, Z sections of 3 µm each (34 sections in total) were taken for 18 min imaging time, every 10 min, for total of 25 cycles. For Supplementary Movie 2, Z sections of 3 µm each (32 sections in total) were taken for 14 min imaging time, every 20 min, for total of 11 cycles. Z stack series were combined to movies in video for Windows using the Zen software (Zeiss).

### Preparation of eye images

Heads of 1-day-old males were disconnected from the body using a scalpel and cut again to separate the two eyes. 20 eyes were examined for each genotype. Images were obtained using a stereo microscope (MZ16F; Leica) connected to a DS-Fi1 camera (Nikon).

### Raeppli experimental design and visualization

Three days after egg laying (AEL), larvae carrying the transgene with the nucleus localized Raeppli tissue labeling tool (which can mark 90% of the cells in a primordium with different combinations of four spectrally separable bright fluorescent proteins^23^), *hs*-FLP, and *sal-*Gal4 constructs, in the background of the *drice*^−/−^ mutant, were exposed to heat-shock at 37 °C for 2 h to induce the generation of multiple color fluorescent clones, through heat-shock-induced phiC31 integrase-based recombination. 5 days AEL, larvae were irradiated (40 Gy, X-ray) to induce ICM. 7 days AEL (48 hpi), WDs were dissected (non-irradiated larvae were dissected at 6 days AEL) and visualized under the LSM780 confocal microscope. The 405 nm channel was omitted from the final images in order to avoid the partially masking autoflorescence of the tracheas.

### Manual quantification of migrating cells

The number of migrating cells in each fixed WD (18 Gy, 48 hpi) were manually counted under the confocal microscope. Cells were counted from the middle of the pouch area proximally to the notum edge. Each biological repeat (3–9 repeats for each genotype, except for *nbs1*^*Ri*^; *drice*^−/−^ for which we performed only one biological repeat, and *atm*^*Ri*^; *drice*^−/−^ and *bsk*^*Ri*^; *drice*^−/−^ for which we performed two biological repeats), had its own *drice*^−/−^ mutant control. The mean score of the *drice*^−/−^ control group was set as 100, and the values of the other genotype groups were expressed as percentage of the control. Migration levels are represented as mean ± SEM of pooled results obtained from the indicated number of samples in each experiment. For *cdc42*^*N17*^; *drice*^−/−^ and its control, the migrating cells were counted only proximally to the pouch area edge, because of the presence of cell clusters near the pouch area.

### Computational quantifications in Fig. 4a

Because of the difficulty to manually count migrating cells that appear in large clusters, such as in the *dcp-1*^−/−^; *drice*^−/−^ double mutant, we applied computational imaging analysis. Fluorescent-positive pixel areas were measured using the ImageJ program (National Institutes of Health; Abramoff et al., 2004) and divided by the corresponding WD areas as follows: for TUNEL, it was divided by the total disc area; for cPARP, it was divided by the Venus expression area (i.e., the WD pouch area); for ICM levels, it was divided by the total area proximal to the pouch. The thresholds for fluorescent-positive pixels were determined manually for TUNEL and cPARP and automatically for ICM levels. WD areas were manually measured using the ImageJ program. Biological repeats were performed twice for TUNEL and five times for cPARP and ICM experiments. The mean score of the WT control groups (for TUNEL and cPARP) and *dcp-1*^−/−^*; drice*^−/−^ double mutant group (for ICM) were set as 100, and the values of the other genotype groups were expressed as percentage of the control groups. TUNEL, cPARP, and ICM levels are represented as the mean ± SEM of pooled results obtained from the indicated number of samples in each experiment.

### Quantification of MMP1 and Venus double-positive cells

Non-irradiated *drice*^−/−^ mutant and *dcp-1*^−/−^; *drice*^−/−^ double mutant WDs stained with the anti-MMP1 antibody were visualized under the confocal microscope. For each WD, 16 Z sections of 3 µm each were taken and the number of MMP1 and Venus double-positive cells was manually counted from the Z stack images. Six biological repeats were performed. Data is represented as the mean ± SEM of pooled results obtained from the indicated number of samples in each experiment.

### Quantification of hemocyte numbers

Fixed WDs with at least one fluorescently-labeled hemocyte were counted. The percentage of the WDs with hemocytes was calculated from the total numbers of counted WDs.

### Quantification of cleaved Dcp-1 staining

*drice*^−/−^ mutant WDs stained with the anti-cleaved Dcp-1 antibody were visualized under the confocal microscope using the 40× lens. For each WD, Z sections of 1 µm each were taken. Migrating cells and the pouch region were segmented automatically using Imaris surface module (version: 9.0.0, by Bitplane) according to the CPV’s Venus expression. Some manual corrections were done to adjust the automatic surface segmentation. The shortest distance from the pouch borders and the average anti-cleaved Dcp-1 staining intensity (measured by average pixel intensity) of each cell were calculated using Imaris. Cells that were found in a distance shorter than 0.5 µm from the pouch were automatically discarded to prevent misidentification between migrating cells and pouch cells. The anti-cleaved Dcp-1 staining intensity vs. the distance from the pouch data was clustered with Euclidian distance clustering and represented by a heatmap using the MATLAB software (version: R2017b, MathWorks).

### Statistical analyses

Statistical analysis for ICM levels (except for Fig. 4a) was performed using a two-way ANOVA accounting for treatment and batch effect using R software (R Core Team, 2016).

Statistical analysis for cPARP and TUNEL in Fig. 4a was performed using a one-way ANOVA (the batch effect was not significant and therefore removed from the ANOVA model) followed by Tukey multiple comparisons post hoc test using STATISTICA (data analysis software system, version 12, StatSoft, Inc. 2013).

Statistical analysis for ICM levels in Fig. 4a was performed using a two-way ANOVA followed by Tukey multiple comparisons post hoc test.

Statistical analysis of WDs with hemocytes was performed using chi-squared test using R software.

Statistical analysis for MMP1 and Venus double-positive cells was performed using a two-tailed unpaired Student’s t test.

Correlation between migration distance and cleaved Dcp-1 intensity of the cells was analyzed using ANCOVA, with WD as a categorical factor, and distance as a continuous factor using R software (R Core Team, 2016). Cleaved Dcp-1 intensity levels were log transformed in order to reduce differences in variances between WDs.

For all the relevant experiments, the residuals were tested for normality using the Shapiro-Wilk test, and differences in variance between groups were tested using Levene’s test. All the results were found valid except for a few acceptable violations.

For all statistical analyses values of *P* < 0.05 were accepted as statistically significant.

Sample size was determined according to our previous experience using this system.

### Image processing

All the Figures were organized using the Adobe Photoshop program. Figure 3, Supplementary Fig. 2 and Supplementary Fig. 5a were first processed using the Imaris program to project the 3D images. For Supplementary Fig. 5a, surfaces were first applied to the pouch area and the migrating cells using the Imaris surface module according to CPV expression.

### Data availability

The authors declare that the data supporting the findings of this study are available within the paper and its supplementary information files.

## Electronic supplementary material

Supplementary Information

Description of Additional Supplementary Files

Supplementary Movie 1

Supplementary Movie 2
