# Supplementary material for: Caspases maintain tissue integrity by an apoptosis-independent inhibition of cell migration and invasion
Source: Nat Commun. 2018 Jul 18;9:2806. doi: 10.1038/s41467-018-05204-6 (PMC6052023; doi:10.1038/s41467-018-05204-6)
Supplement: Supplementary file 1 — Supplementary Information [file 41467_2018_5204_MOESM1_ESM.pdf]

Caspases maintain tissue integrity by an apoptosis-independent inhibition of cell migration and invasion

Gorelick-Ashkenazi et al.

Supplementary Information

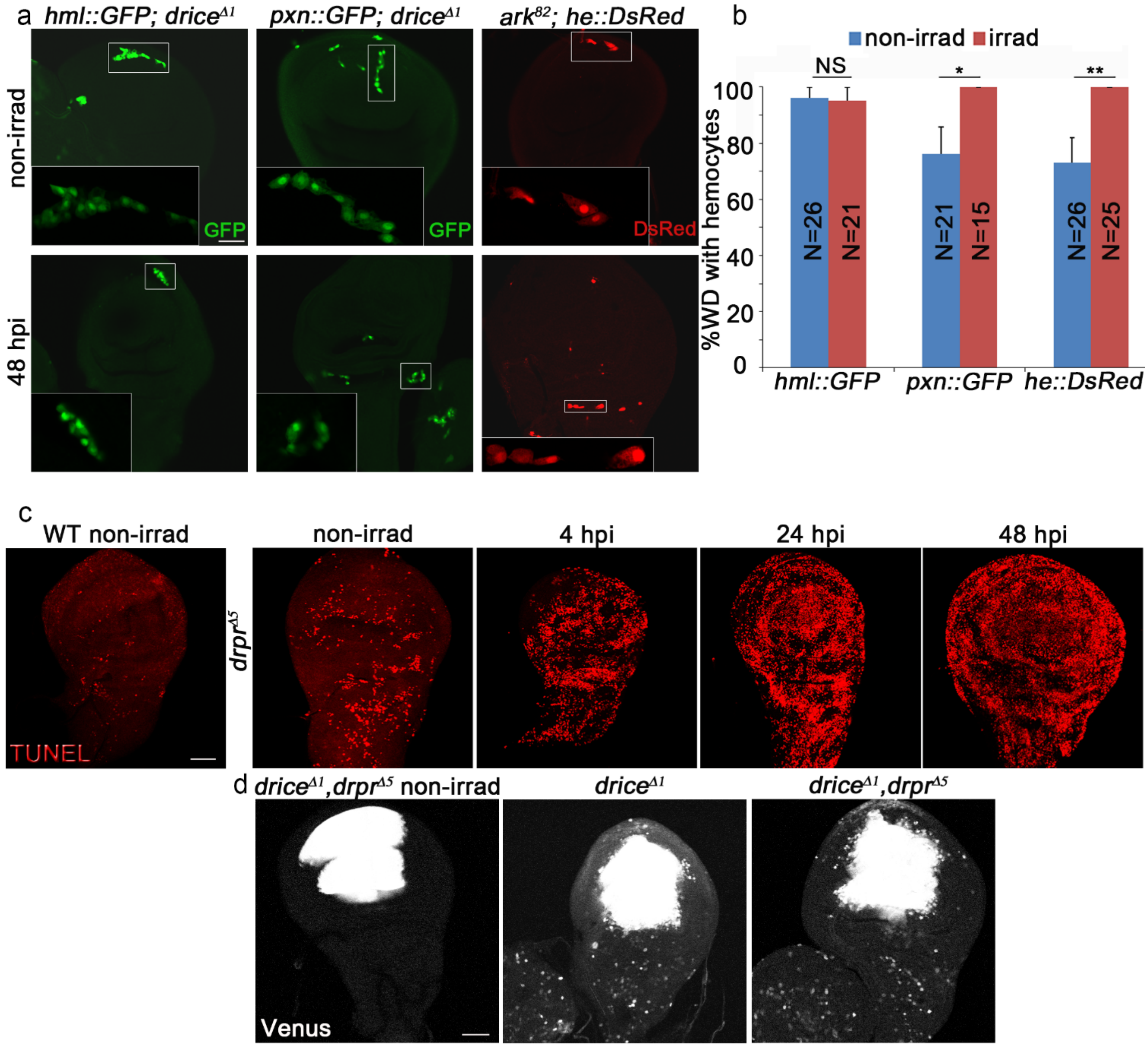

Supplementary Figure 1 Cell migration during ICM is not mediated by professional phagocytes (hemocytes). **a**, Hemocyte cell shape, numbers, and migration pattern do not change upon ICM induction. Representative images of *drice*$^{-/-}$ and *ark*$^{-/-}$ WDs expressing 3 different hemocyte markers, *hemolectin* (*hml*), *peroxidasine* (*pxn*) or *hemese* (*he*), untreated and treated with irradiation (50 Gy) to induce ICM. The insets contain higher magnification images of the areas marked by rectangles. **b**, Irradiation induces no to very low increase in the number of WDs with hemocytes. Error bars represent s.e.m. *$P < 0.05$, **$P < 0.01$, chi-squared test. NS, non-significant. **c**, Irradiated (50 Gy) WDs lacking the major engulfment receptor (Drpr) do not display the stereotypic cell corpse clearance pattern shown in Fig. 1b. **d**, ICM is not attenuated in irradiated (50 Gy) *drice*$^{-/-}$ WDs lacking Drpr and examined at 48 hpi. Scale bars in (**a**), (**c**), and (**d**), 50 μm.

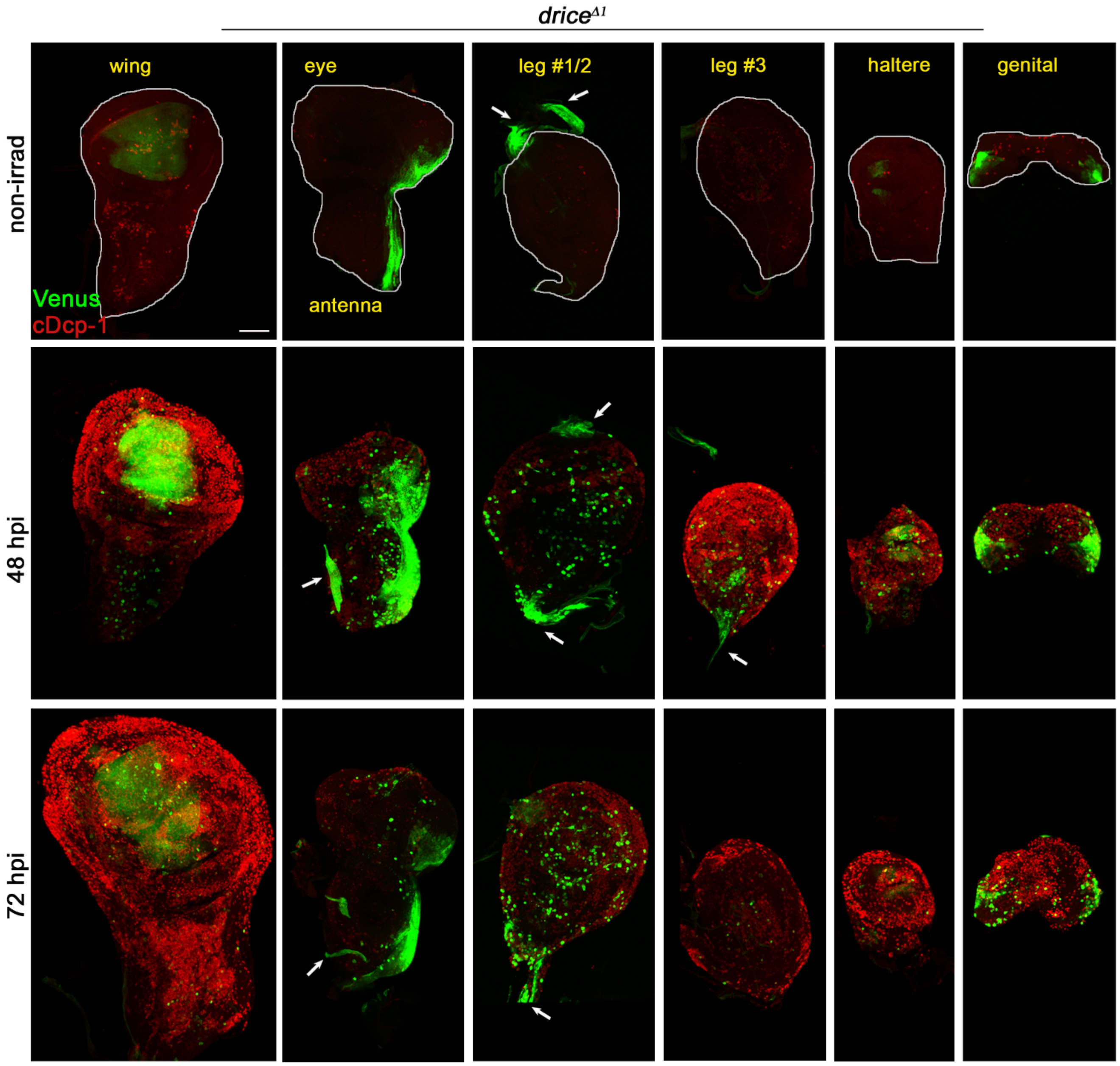

Supplementary Figure 2 Irradiation-induced migrating cells are essentially found in all the imaginal discs. Representative high-resolution 3D projection images of non-irradiated and irradiated (40 Gy, 48 and 72 hpi) *drice*$^{-/-}$ mutant imaginal discs expressing the CPV (Venus; green) from the *sal*-Gal4 driver. The imaginal discs were also stained to reveal activated Dcp-1 (cDcp-1; red), which validates the irradiation treatment. White arrows indicate tracheal autofluorescence. Migrating cells are never detected in non-irradiated imaginal discs, whereas following irradiation, numerous migrating cells are detected in all the discs, including the leg imaginal discs that do not express the *sal*-Gal4 driver. Scale bar, 50 µm.

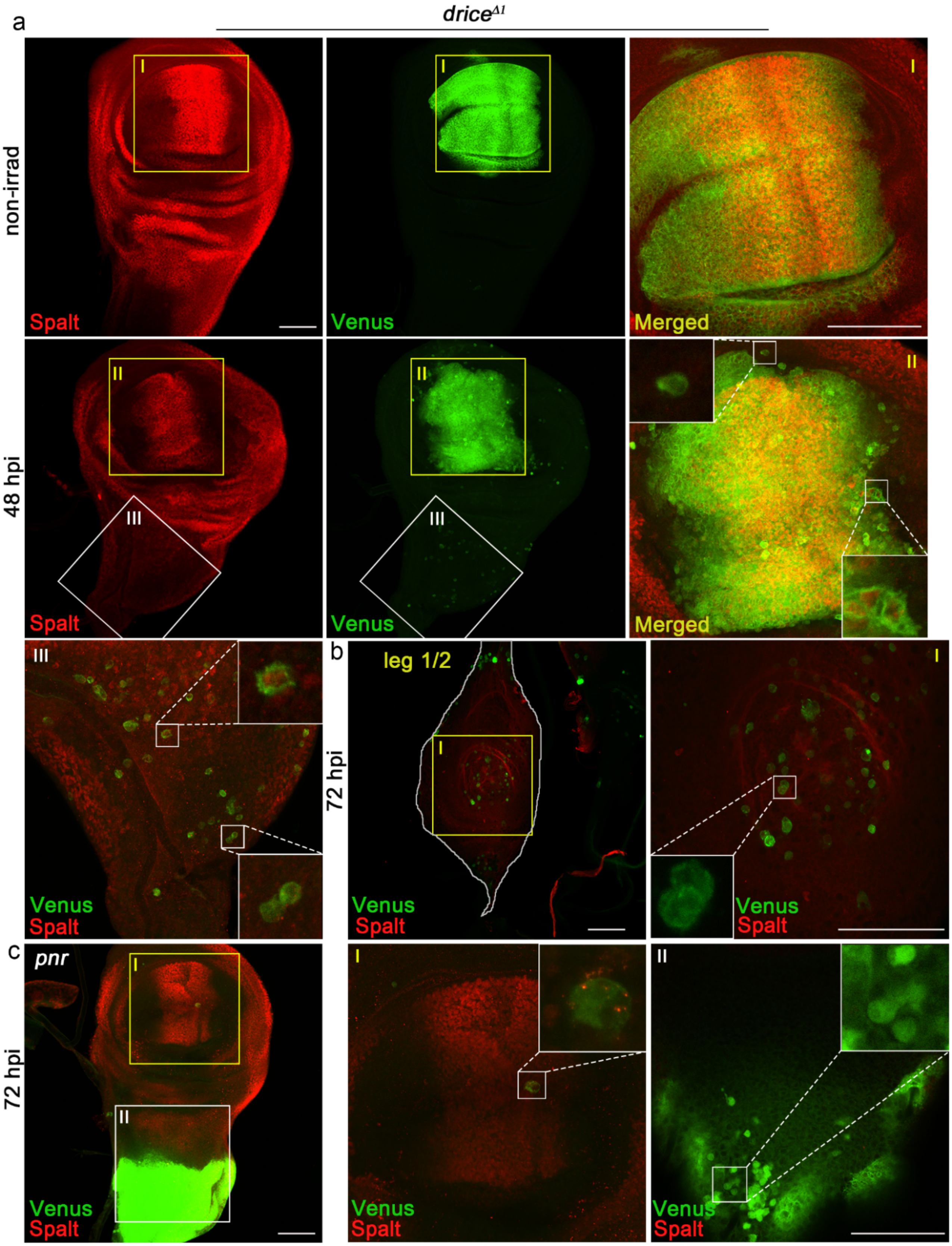

Supplementary Figure 3 Endogenous Spalt expression is not prerequisite for ICM induction. Maximal intensity projections of *drice*$^{-/-}$ mutant wing (**a, c**) and leg (**b**) imaginal discs were stained to visualize the endogenous Spalt protein (red). The CPV reporter (Venus; green) was expressed using the *sal*-Gal4 (**a, b**) or the *pnr*-Gal4 (**c**) drivers. **a,** Non-irradiated and irradiated WDs (40 Gy, 48 hpi) show differential

expression of Spalt throughout the tissue. Note that endogenous Spalt expression in the pouch is narrower than the *sal*-Gal4/CPV expression. **a, I, II, III,** These images are enlargements of the corresponding square areas in (**a**). **a, II, III,** Migrating cells that whether or not express Spalt are both detected throughout the WD. Magnifications of some individual cells appear in the white insets. **b,** Endogenous Spalt is not expressed in the leg imaginal discs and in at least most of the migrating cells that invaded this tissue following ICM induction (40 Gy, 72 hpi). **b, I,** This image is an enlargement of the corresponding square area in (**b**). The inset contains a higher magnification image of individual migrating cells that appear in the area marked by a white square. **c,** Irradiation-induced migrating cells from the WD notum do not display Spalt expression, even when they reach the Spalt-expressing pouch area. **c, I,** This image is an enlargement of the corresponding square area in (**c**). **c, II,** This image is an enlargement of a single optical section in the corresponding square area in (**c**), showing the migrating cells originated in the WD notum. The insets contain higher magnification images of individual migrating cells that appear in the areas marked by white squares. Scale bars in (**a-c**), 50 µm.

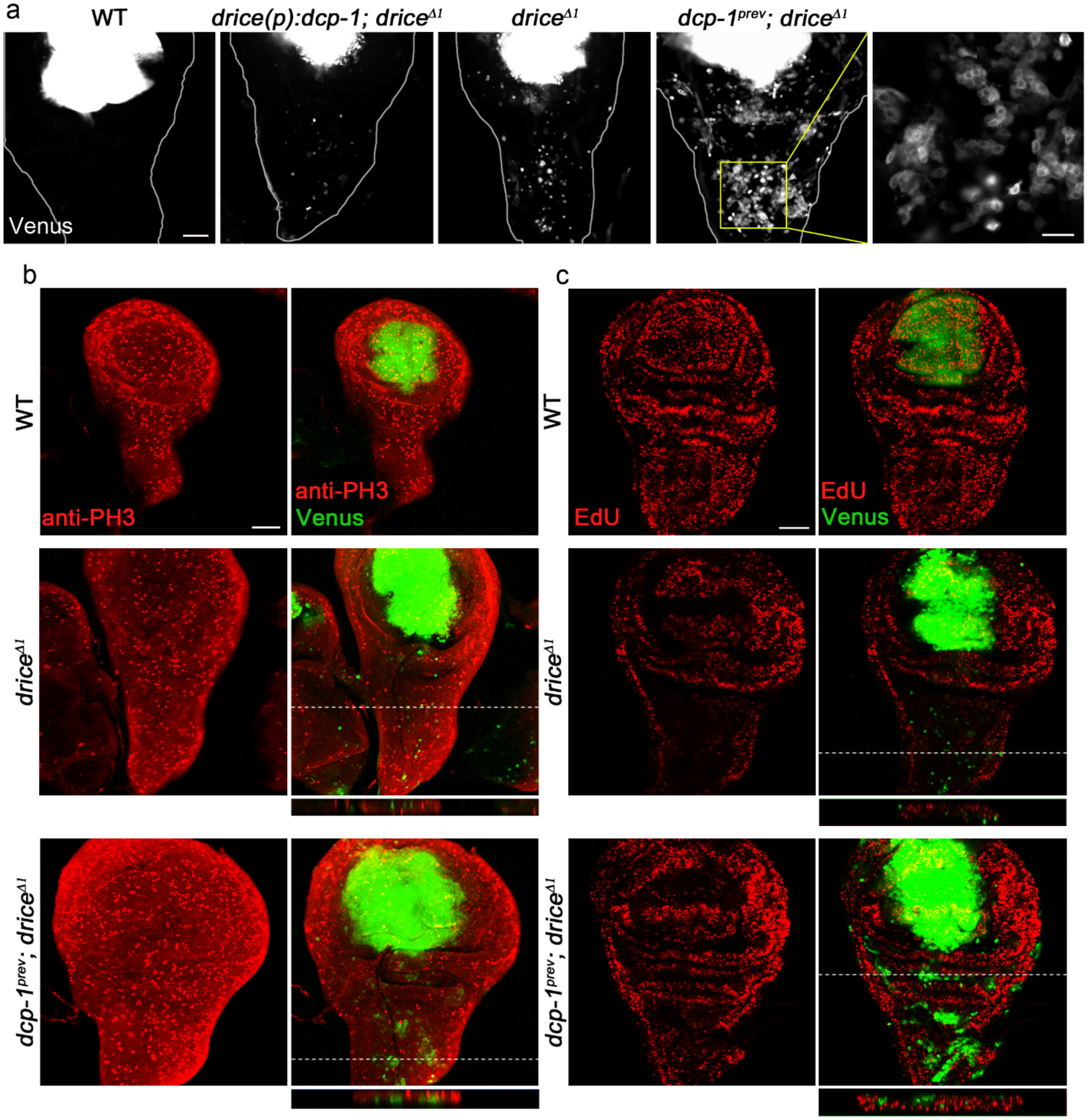

Supplementary Figure 4 ICM levels inversely correlate with the cell apoptotic potential. **a**, Representative images of WDs from the indicated genotypes treated with a dose of 40 Gy X-irradiation and visualized 48 hpi. Scale bar, 50 µm. The yellow square marks an area with numerous clusters of migrating cells generated when the effector caspase activity is completely compromised. The image on the right is a higher magnification of the area indicated by the square; scale bar, 20 µm. **b**, **c**, Migrating cells (green) and dividing cells (red) are largely mutually exclusive. Representative images of WD optical sections (at the strongest signal) from the indicated genotypes at 48 hpi, treated either with γ-irradiation (50 Gy) and stained with an anti-phosphohistone H3 antibody (anti-PH3, red; **b**), or X-irradiation (40 Gy) and labeled with EdU (red; **c**). Dashed lines indicate the regions for which Z-axis projections (bottom) were generated. Scale bars, 50 µm.

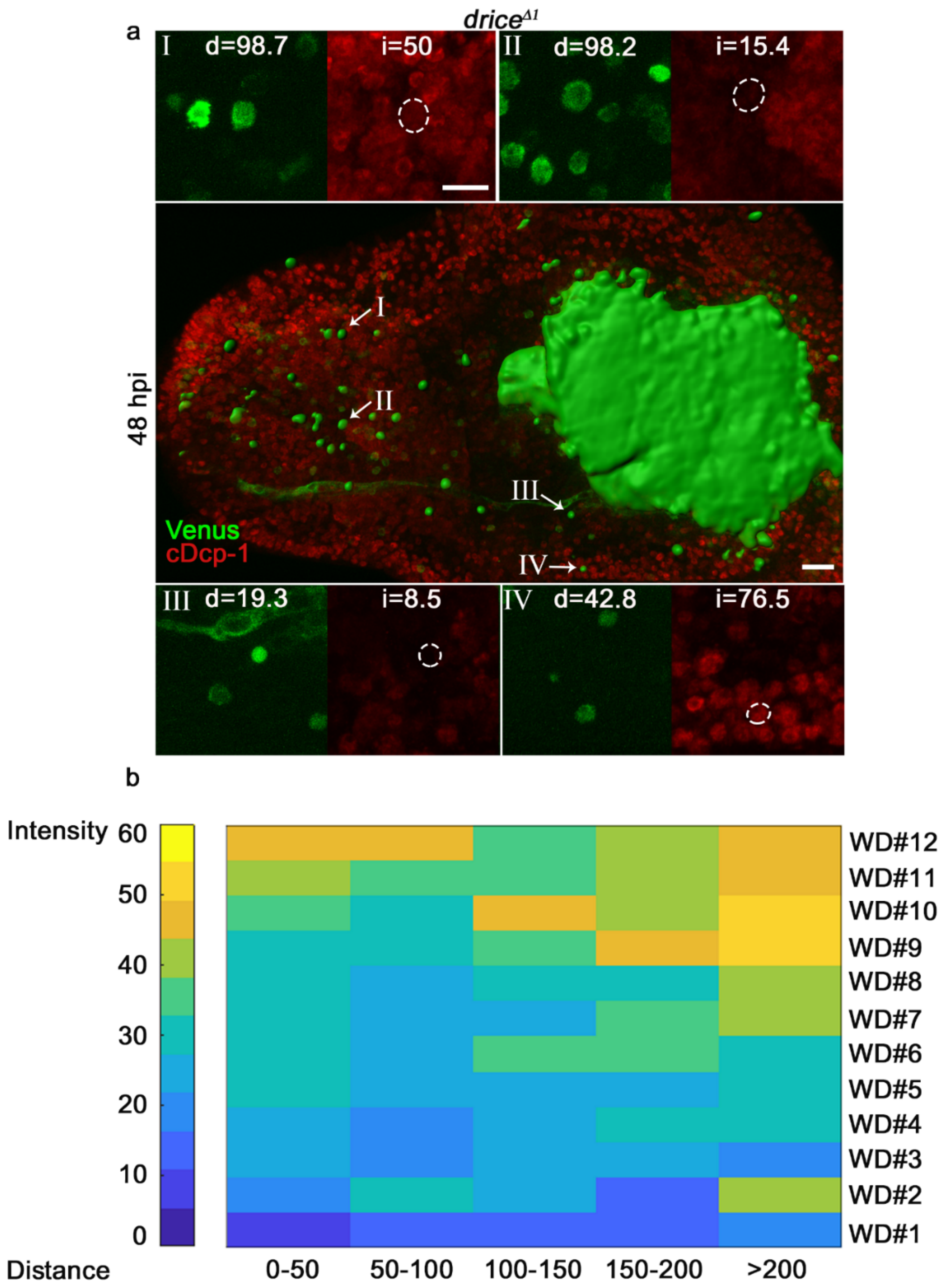

Supplementary Figure 5 The Dcp-1 activity level is not correlated to the distance of the migrating cells from their expression domain. **a,** A representative 3D projection image depicting one out of 12 *drice*$^{-/-}$ mutant WDs, irradiated (40 Gy, 48 hpi) to induce ICM. This WD expresses the CPV reporter (Venus; green) from the *sal*-Gal4 driver and stained to reveal activated Dcp-1 (cDcp-1; red). No correlation was found between migration distance from the *sal*-Gal4 expression domain and cDcp-1 staining levels (N=12; adjusted R^2=0.28). Magnification images of individual migrating cells that covered various distances appear above and below the main image, and correspond to the indicated Roman numerals. d, the shortest distance from the WD pouch border in µm; i, cDcp-1 fluorescence intensity measured by the average pixel intensity of each cell. Scale bars in the main image, 20 µm and in (**I-IV**), 10 µm. **b,** A heatmap of the 12 WDs (y-axis), displaying the average cDcp-1 fluorescence intensities (color-coded) in migrating cells grouped according to five distance categories (µm; x-axis).

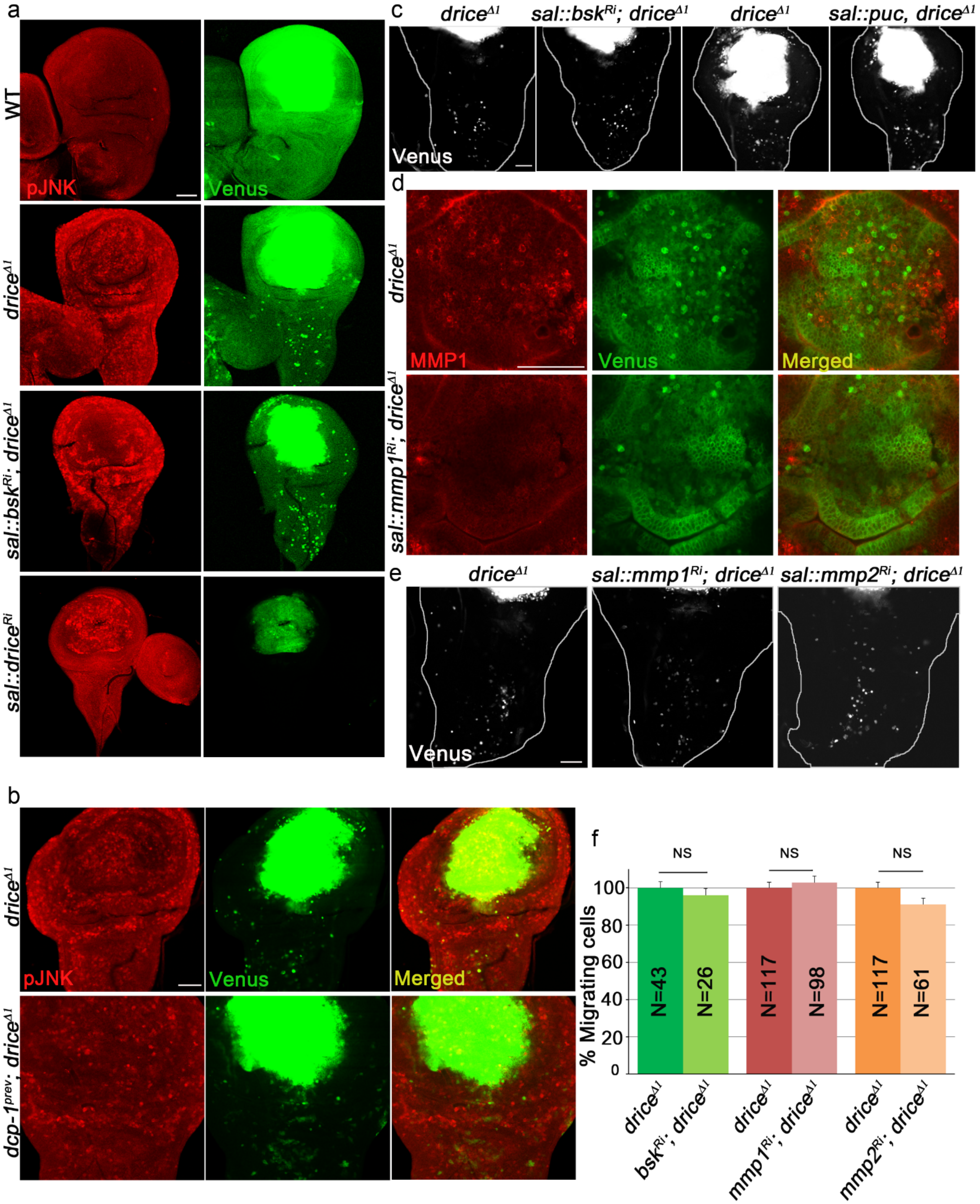

Supplementary Figure 6 JNK and MMP1 are not essential for ICM induction. **a**, JNK activity (phospho-JNK; pJNK; red) is induced in the *drice*$^{-/-}$ mutant WD and in the *drice* knockdown area (WD pouch), but not in WT WDs at 48 hpi (50 Gy). Note, that the specificity of the RNAi against *jnk* (*bsk*^{Ri}) was validated. **b**, Induction of pJNK expression is significantly reduced in the *dcp-1*$^{-/-}$*; drice*$^{-/-}$ double mutant background as compared to the *drice*$^{-/-}$ mutant WDs (40 Gy, 48 hpi). **c**, Knockdown of *jnk* (*bsk*^{Ri})

and overexpression of the JNK inhibitor Puckered (Puc) in the pouch area do not attenuate ICM in the *drice*$^{-/-}$ mutant (48 hpi, 18 Gy or 50 Gy, respectively). See also the quantification of ICM level in the *bsk*^{RI} WDs in (**f**). **d**, Validation of the specificity of the RNAi against *mmp1* (*mmp1*^{Ri}). Shown are the *drice*$^{-/-}$ mutant WD pouch regions stained with anti-MMP1 antibody (red) at 48 hpi (50 Gy). **e**, Knockdown of *mmp1* and *mmp2* in the pouch area do not attenuate ICM in the *drice*$^{-/-}$ mutant (48 hpi, 18 Gy). **f**, Quantifications of ICM levels in WDs following pouch area knockdowns of *jnk* (*bsk*^{RI}; **c**), *mmp1* and *mmp2* (**e**) in the *drice*$^{-/-}$ mutant background. The quantifications are presented as averages of the migrating cell numbers relative to control. The examined WD numbers (N) are indicated for each graph bar. Error bars indicate s.e.m. NS, non-significant, two-way ANOVA. Scale bars, 50 µm.

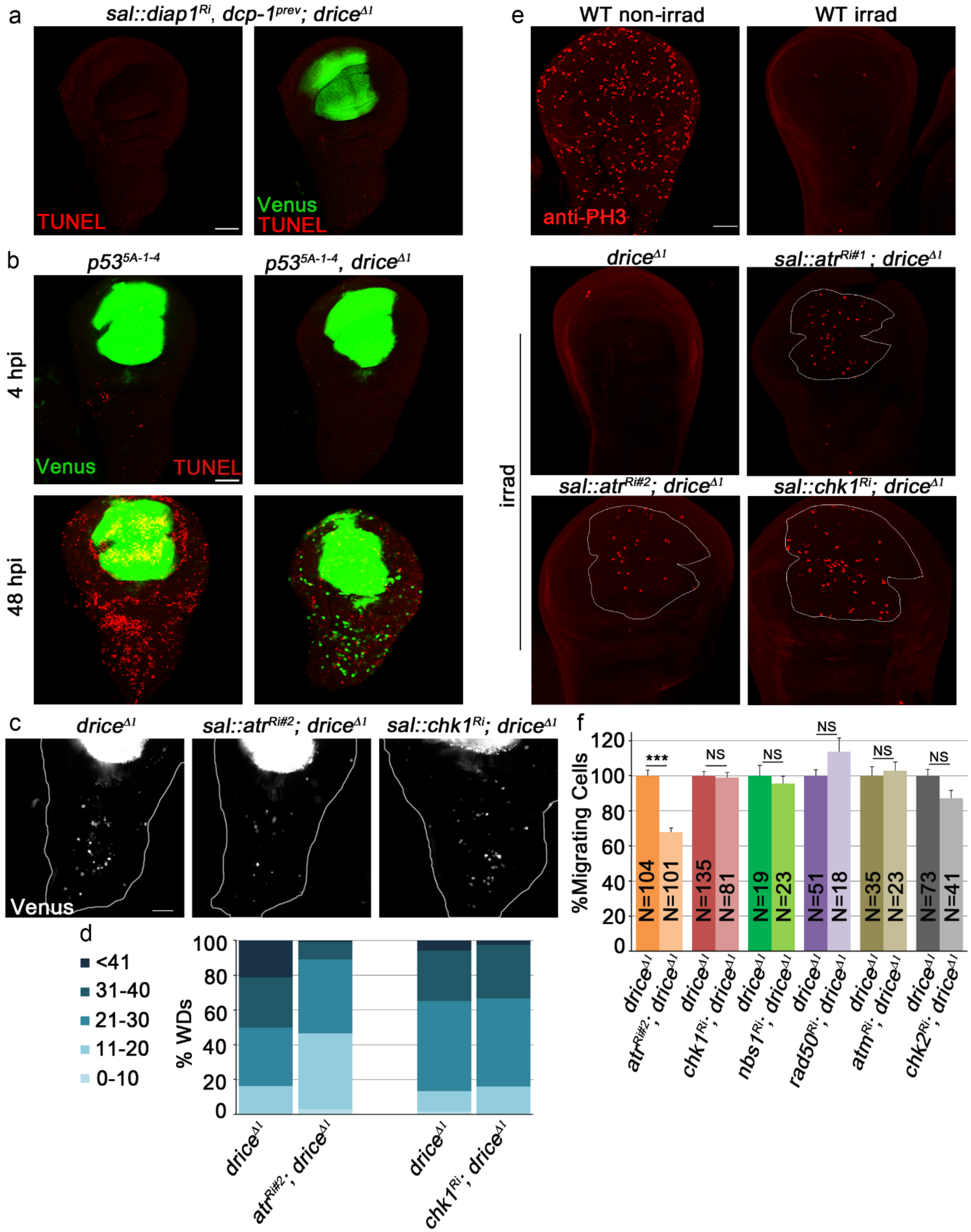

Supplementary Figure 7 ICM is induced upstream of p53 and the core apoptotic machinery through the 9-1-1/ATR branch, not the MRN/ATM/Chk2 branch, of the DNA damage response pathway. **a**, Pouch-specific knockdown of *diap1* does not induce ICM in non-irradiated *dcp-1*$^{-/-}$; *drice*$^{-/-}$ double mutant WDs. **b**, ICM is not attenuated in irradiated WDs (50 Gy) from *p53*$^{-/-}$, *drice*$^{-/-}$ double mutant

flies. **c**, **d**, Pouch-specific knockdown of *atr* but not its best-studied effector, *chk1*, attenuates ICM. Selected images of irradiated WDs (18 Gy, 48 hpi) of the indicated genotypes following ICM induction (**c**) and the corresponding quantifications presented as the percentage of WDs with each of 5 different migrating cell number categories (color coded by different shades of blue; **d**). **e**, Pouch-specific knockdowns of *atr* and *chk1* overcome cell cycle checkpoint arrest in irradiated WDs (40 Gy, 1-2 hpi). These fly lines (which have the *drice*$^{-/-}$ background) were also tested for effects on ICM (**c**, **d**, **f** and Fig. 6a-c). Scale bars in (**a**-**c**, **e**), 50 µm. **f**, Quantifications of ICM levels (18 Gy, 48 hpi) following pouch-specific knockdowns of the indicated components in the MRN/ATM/Chk2 pathway. The *atr* and *chk1* knockdowns serve as positive and negative controls, respectively. The examined WD numbers (N) are indicated for each graph bar. Error bars represent s.e.m. ****P* < 0.001. NS, non-significant, two-way ANOVA, for *nbs-1*^{Ri} two-tailed unpaired Student’s t test.

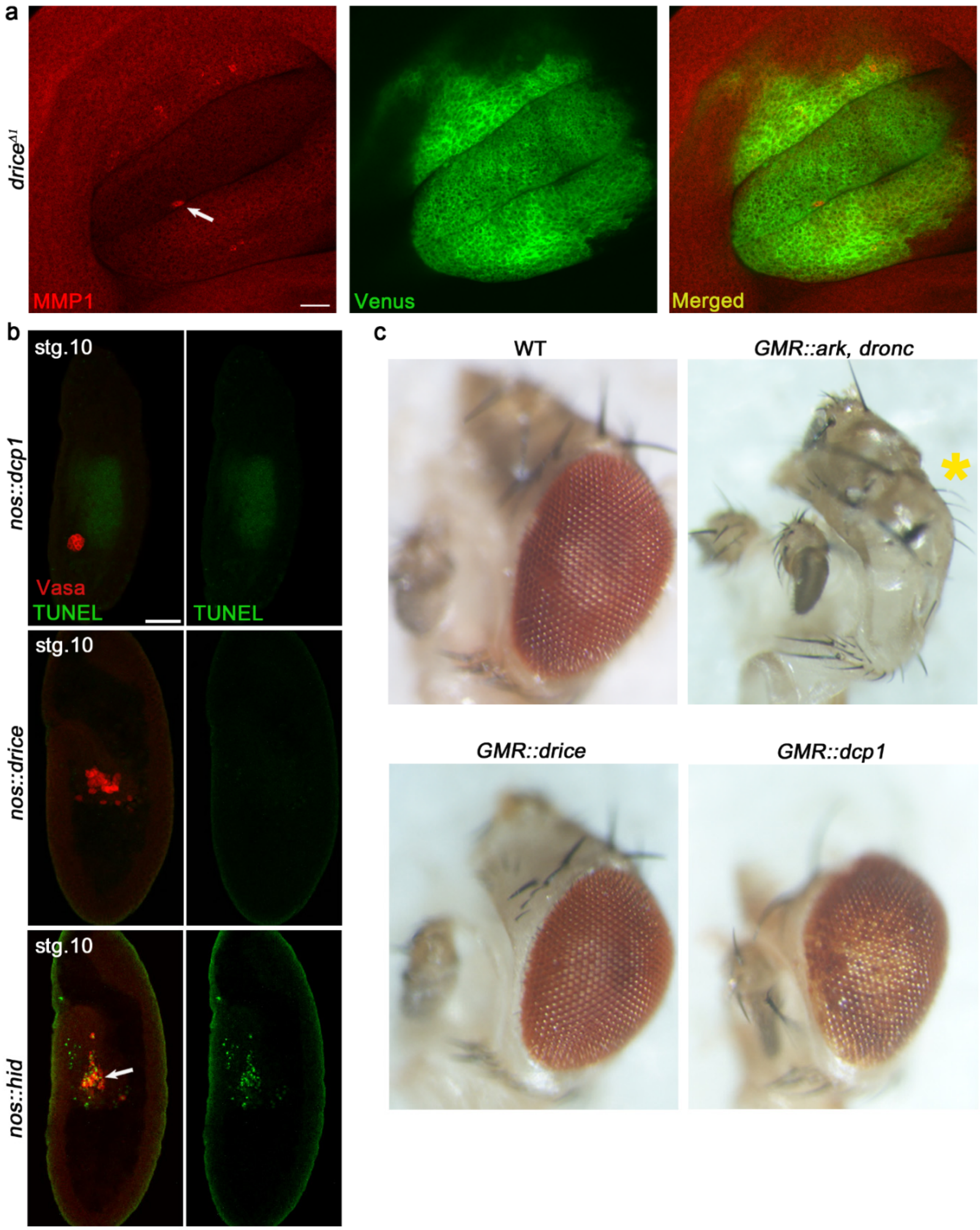

Supplementary Figure 8 Low effector caspase levels that are sufficient to inhibit cell migration are below the threshold required to trigger apoptosis. **a**, A representative image of the pouch area of a non-irradiated *drice*$^{-/-}$ WD stained for MMP1 expression. The pouch cells are marked in green (Venus) by the *sal*::CPV. The arrow indicates a spontaneously emerged, MMP1-positive, delaminating/migrating cell. Scale bar, 20 µm. **b**, **c**, Overexpression of the full-length cDNAs of *dcp-1* and *drice* does not induce apoptosis in stage 10 embryo PGCs (red, **b**) or the adult eye (**c**). The arrow in (**b**) indicates dying PGCs (green) overexpressing the pro-apoptotic gene *hid*. The asterisk in (**c**) indicates the remaining head capsule after the eye ablation (due to apoptosis) induced by the simultaneous overexpression of the apoptosome components. Scale bar in (**b**), 50 µm.

Supplementary Discussion

A previous paper by Cagan and colleagues proposed an opposite effect of caspases on cell migration, suggesting that low caspase activity levels can promote JNK-dependent (local) cell invasion in *Drosophila* WDs[1]. The differences in the conclusions between our study of ICM and Cagan's described phenomenon could be attributed to the different setups of the two experimental systems. In particular, for the Cagan's phenomenon, apoptosis was induced at the level of the core apoptotic machinery (i.e. by overexpressing Hid or an active form of Dronc)[1], whereas in the current work, we triggered apoptosis much upstream in the signaling cascade, namely by irradiation. Indeed, as opposed to the Cagan's phenomenon which requires JNK activity, we showed that JNK activity is dispensable for ICM induction, which instead requires the DNA-damage response pathway induced by irradiation. Furthermore, the migration level and invasive potential triggered by irradiation are much more dramatic and pronounced than the more local/confined effects described by Cagan and colleagues. These local effects exhibited in the Cagan's phenomenon may imply that the cells are passively migrating within local professional phagocytes, which may also explain the requirement for caspase activity in this system (to produce the 'find me' and 'eat me' signals).

Another interesting phenomenon detected in specific *Drosophila* epithelial tissue setups and termed cell mixing, was recently described by Moreno and colleagues to promote invasiveness and destruction of healthy tissues[2]. However, comparison between ICM and cell mixing characteristics strongly suggests that these are two distinct phenomena. First, the hypothesis that caspases might target cytoskeleton remodeling proteins to facilitate cell mixing is in contrast to the ICM induction model, indicating that caspases inhibit, rather than facilitate, ICM induction. Secondly, the setup which triggers ICM is different from the setup which induces cell competition (and consequent cell mixing), as the latter requires two defined populations of "winner" and "loser" cells. In contrast, the most efficient cell migration/invasion during ICM requires that the entire cell population in the tissue be compromised for caspase activity, and that the whole animal is irradiated (i.e. all the cells receive the signal for apoptosis). Thirdly, cell mixing mainly occurs locally over short distances, whereas ICM is a long-distance phenomenon, in which cells also invade other tissues. Fourthly, the "loser" cell morphology during cell mixing[2] supports passive displacement by the "winner" cells, rather than displaying rounded and condensed morphology with dynamic protrusions, which characterize the irradiation-induced actively migrating cells. Lastly, even in cases when ICM was induced only in a restricted WD area, the vast majority of migrating cells remained confined to the caspase-compromised area, and only rarely invaded the surrounding WT tissue. This is in contrast to cell mixing, which is induced by junction remodeling and cell-cell intercalation[2].

Supplementary References

1. Rudrapatna,V.A., Bangi,E., & Cagan,R.L. Caspase signalling in the absence of apoptosis drives Jnk-dependent invasion. *EMBO Rep.* **14**, 172-177 (2013).
2. Levayer,R., Hauert,B., & Moreno,E. Cell mixing induced by myc is required for competitive tissue invasion and destruction. *Nature* **524**, 476-480 (2015).
