# Supplementary material for: Caspases maintain tissue integrity by an apoptosis-independent inhibition of cell migration and invasion
Source: Nat Commun. 2018 Jul 18;9:2806. doi: 10.1038/s41467-018-05204-6 (PMC6052023; doi:10.1038/s41467-018-05204-6)
Supplement: Supplementary file 2 — Description of Additional Supplementary Files [file 41467_2018_5204_MOESM2_ESM.docx]

**Description of Additional Supplementary Files**

File Name: Supplementary Movie 1

Description:

**Live imaging of cells migrating away from the WD pouch area following ICM induction.** Irradiated (50 Gy) drice-/- WDs were dissected at 33 hpi, cultured for 14 additional hours, during which they were imaged (for the last 12 hours) under the confocal microscope. Migrating cells (green, Venus) are labeled by the sal::CPV. Scale bar, 50 µm.

File Name: Supplementary Movie 2

Description:

**Live imaging of migrating cells proximal to the pouch area following ICM induction.** Irradiated (50 Gy) drice-/- WDs were dissected at 48 hpi, cultured for 8 additional hours, during which they were imaged (for the last 6 hours) under the confocal microscope. Migrating cells (green, Venus) are labeled by the sal::CPV. Scale bar, 50 µm.
